# Pallado-Catalyzed Cascade Synthesis of 2-Alkoxyquinolines from 1,3-Butadiynamides

**DOI:** 10.3390/molecules29153505

**Published:** 2024-07-26

**Authors:** Illia Lenko, Alexander Mamontov, Carole Alayrac, Bernhard Witulski

**Affiliations:** Laboratoire de Chimie Moléculaire et Thio-organique (LCMT), CNRS UMR 6507, ENSICAEN, Université de Caen, Normandie Univ, 6 Bd Maréchal Juin, 14050 Caen, France

**Keywords:** 1,3-diynamides, ynamides, [4]cumulenimines, homogeneous catalysis, heterocycles, annulation reactions, cascade reactions, quinolines

## Abstract

A novel synthesis strategy to access 2-alkoxyquinoline derivatives via a palladium-driven cascade reaction is disclosed. Unlike classic methods based on the alkylation of 2-quinolones with alkyl halides, the present method benefits from the de novo assembly of the quinoline core starting from 1,3-butadiynamides. Palladium-catalyzed reaction cascades with *N*-(2-iodophenyl)-*N*-tosyl-1,3-butadiynamides and primary alcohols as external nucleophiles proceed under mild reaction conditions and selectively deliver a variety of differently functionalized 4-alkenyl 2-alkoxyquinolines in a single batch transformation.

## 1. Introduction

**Figure 1:**
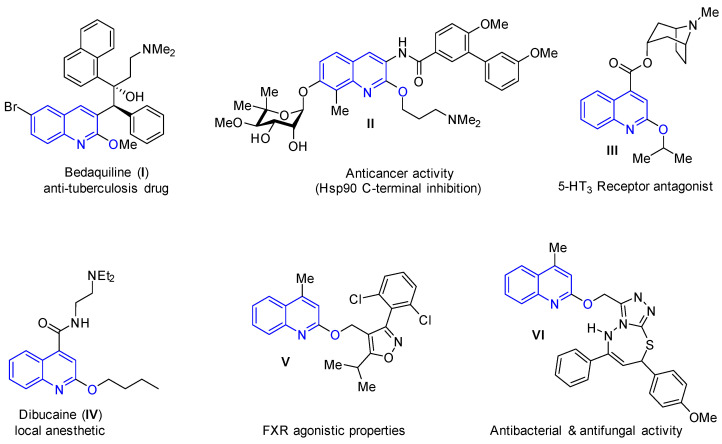
Examples of biologically relevant 2-alkoxyquinoline derivatives.

The quinoline motif is found in the molecular structure of a large number of drugs, pharmaceuticals and natural products with relevant biological activities [1,2,3,4]. Therefore, quinolines serve as a privileged molecular platform for the development of new therapeutic agents. Within these, several differently functionalized 2-alkoxyquinolines have been reported (Figure 1):

For instance, bedaquiline **I** has been approved by the U.S. Food and Drug Administration for the treatment of drug-resistant tuberculosis [5]. 2-alkoxyquinoline **II** belongs to a set of novobiocin analogs where the 2-alkoxyquinoline replaces the original coumarin core. **II** displays anticancer activity by targeting the Hsp90 (heat-shock protein 90) C-terminal region (Figure 1) [6]. A series of 2-alkoxyquinolines bearing a lipophilic group at the 1- or 2-position was tested as new 5-HT_3_ receptor antagonists, and 2-alkoxyquinoline **III** revealed one of the highest affinities (*K*i = 0.31 nM) [7]. 2-*n*-butyloxyquinoline dibucaine **IV** is a local anesthetic [8]. Furthermore, 2-alkoxyquinoline **V** functions as a nonsteroidal agonist of the farnesoid X receptor (FXR) [9], and 2-alkoxyquinoline **VI** is an antibacterial agent [10].

2-Alkoxyquinolines also play a crucial role as ligands in palladium-catalyzed C(sp^3^)–H bond functionalization, notably by enabling primary and secondary C(sp^3^)–H arylation [11,12], as well as γ-C(sp^3^)–H acetoxylation of triflyl-protected amines [13]. 

Typically, the synthesis of 2-alkoxyquinolines relies on the modification of suitable quinoline derivatives such as 2-chloroquinolines or 2-quinolones. S_N_Ar reactions of 2-chloroquinolines with sodium alkoxides in alcohol deliver 2-alkoxyquinolines after several hours of heating under reflux [14]. Shorter reaction times of a few minutes were reported under microwave irradiation and in polar solvents such as HMPTA (hexamethylphosphortriamide) or NMP (*N*-methylpyrrolidone) [15]. Another classic synthesis method involves base-mediated *O*-alkylations of 2-quinolones by alkyl halides [6]. However, 2-quinolones are ambident nucleophiles due to their two tautomeric forms: 2-quinolone and 2-hydroxyquinoline. Here, the control of selectivity of *O*- versus *N*-alkylation is a non-negligible issue. Typically, *O*-alkylated products are obtained through the use of a stoichiometric amount of silver salts (Ag_2_CO_3_) [16,17]. The preferential interaction of the silver cation with the N atom ensures blocking of the *N*-site and liberates the *O*-nucleophilic site for *O*-alkylations. Recently, chemoselective Pd-catalyzed *O*-benzylations of 2-quinolones were achieved without the need of stoichiometric amounts of silver salts [18]. This process relies on the use of XantPhosPdCl_2_ as the pre-catalyst and the generation of a phosphine mono-oxide Pd(II) *η*^1^-benzyl complex as the key intermediate. However, the method is not applicable to alkyl halides other than benzyl halides. Whereas a variety of annulation reactions involving nonconventional bond-forming reactions have been developed for functionalized 2-quinolones [19,20], direct routes to 2-alkoxyquinolines based on the assembly of the quinoline core are rare. As an example, Brandsma described the synthesis of 2-*O*-silylated quinolines resulting from the reaction of phenyl isocyanate with lithiated allenes [21]. More recently, the DMAP-catalyzed synthesis of tert-butylquinolin-2-yl carbonates from 2-alkenylanilines and di-tert-butyl dicarbonate was reported [22].

**Scheme 1:**
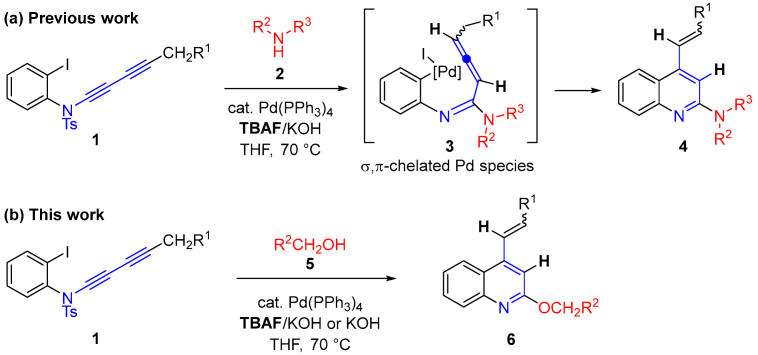
(**a**) Pd-catalyzed cascade reaction of 1,3-butadiynamides **1** with primary or secondary amines **2** leading to 2-amino-4-alkenyl quinolines **4**. (**b**) Pd-catalyzed cascade reaction of 1,3-butadiynamides **1** with primary alcohols **5** yielding 2-alkoxy-4-alkenyl quinolines **6**.

Within our research on the chemistry of ynamides [23,24,25,26,27,28] and 1,3-butadiynamides [29,30,31], we recently disclosed a straightforward route to functionalized 2-amino-4-alkenylquinolines **4** from 1,3-butadiynamides **1** based on a palladium-catalyzed reaction cascade with primary or secondary amines **2** as external nucleophiles (Scheme 1a) [32]. 

The presence of TBAF (tetrabutylammonium fluoride) and KOH induces the cleavage of the tosyl group located at the ynamide nitrogen atom and ensures the involvement of both triple bonds in the annulation reaction cascade via the postulated σ,π-chelated Pd-species **3** as the key intermediate.

Extending the scope of this Pd-catalyzed cascade reaction is an attractive aim. When using readily available alcohols as nucleophiles, it would likely lead to a new approach to versatile 2-alkoxyquinolines via straightforward annulation reactions. We report here that primary alcohols **5** can indeed serve as suitable nucleophiles and reaction partners to convert readily accessible 1,3-butadiynamides **1** into functionalized 4-alkenyl 2-alkoxyquinolines **6** within a single batch and under mild conditions (Scheme 1b).

## 2. Results and Discussion

**Scheme 2:**
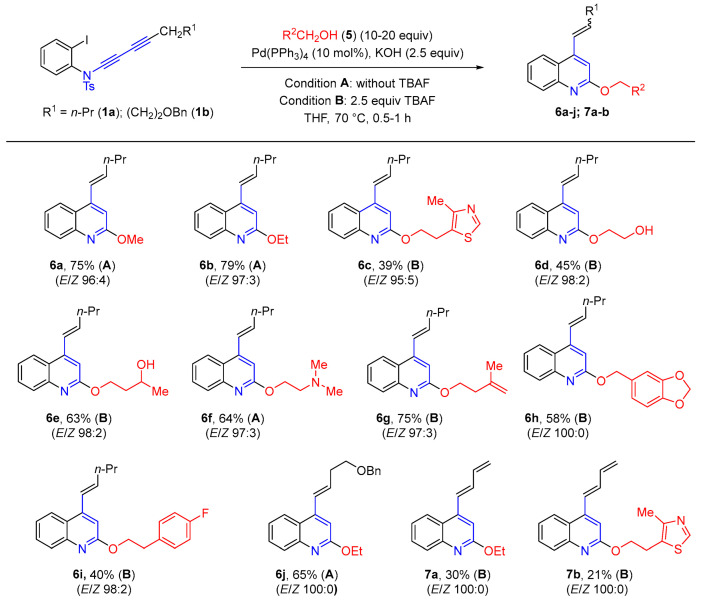
Synthesis of 2-alkoxy-4-alkenylquinolines **6** and 2-alkoxy-4-dienylquinolines **7a,b**.

In our work related to the synthesis of 2-aminoquinolines **4**, where primary or secondary amines served as nucleophiles, TBAF was added to the reaction to improve the solubility of KOH in THF through anion metathesis. Considering that the solubility of KOH is enhanced in methanol, the reaction of 1,3-butadiynamide **1a** (R^1^ = *n*-Pr) was attempted without TBAF. Fortunately, diynamide **1a** was now fully converted into the expected 4-alkenyl 2-methoxyquinoline **6a** in the presence of Pd(PPh_3_)_4_ (10 mol%) and KOH (2.5 equiv) in methanol at 70 °C (Scheme 2). The 2-methoxyquinoline **6a** was obtained in 75% yield with an (*E*)/(*Z*) ratio of 96:4 after column chromatography. Similarly, the 2-ethoxy analogue **6b** was obtained in 79% isolated yield with an (*E*)/(*Z*) ratio of 97:3 when the reaction was carried out in EtOH as the solvent (Scheme 2). Notably, the yield of isolated **6b** was not affected when the amount of EtOH was reduced from 165 to 10 equivalents and using THF as the solvent.

The option for utilizing more sophisticated alcohols was evaluated with 4-methyl-thiazol-5-yl-ethanol (**5c**). No reaction occurred between **1a** and alcohol **5c** (10 equiv) in THF after 1 h of heating at 70 °C and recovering the starting material **1a**. This result might be due to the lack of solubility of KOH in the reaction medium. The addition of EtOH or MeOH to solubilize KOH could not be considered as these might compete as nucleophiles in this reaction. Therefore, the use of TBAF/KOH to induce an anion metathesis was again attempted. The reaction of **1a** (0.11 mmol) with alcohol **5c** (20 equiv) was performed in 1 mL of a commercially available 1M solution of TBAF in THF (9.5 equiv) in the presence of 10 mol% of Pd(PPh_3_)_4_ and 2.5 equivalents of KOH. Gratifyingly, under these modified reaction conditions, the expected 2-alkoxyquinoline **6c** was obtained in 39% yield of the isolated material after 1 h at 70 °C (Scheme 2). The scope of this new 2-alkoxyquinoline synthesis was further studied using 10 mol% of Pd(PPh_3_)_4_, 2.5 equiv of KOH and 2.5 equiv of TBAF in THF as the standard reaction conditions. When glycol (**5d**) was used as the *O*-nucleophile, the product of mono-addition **6d** was the sole product obtained (45% yield). The reaction was chemoselective with 1,3-butanediol (**5e**). Here, the primary alcohol reacted exclusively to give the corresponding 2-alkoxyquinoline **6e** in 63% yield. It is worthy of note that access to quinolines **6d** and **6e** is not as straightforward using classic methods. Reactions with secondary or tertiary alcohols (i.e., cyclopentanol and tert-butanol, respectively) failed, probably due to steric reasons. On the other hand, alcohols tethered to an amino group, an olefin or an electron-rich or electron -poor aryl group were successfully engaged in this new and unprecedented 2-alkoxyquinoline synthesis and yielded a set of 2-alkoxy-4-alkenyl quinolines **6f-i** in 40 to 75% yields of the isolated compounds (Scheme 2). In all cases, the (*E*) isomer was preferentially formed with an (*E*)/(*Z*) ratio higher than 95:5. The reaction of diynamide **1b** (R^1^ = (CH_2_)_2_OBn) in EtOH as the solvent selectively led to the (*E*) isomer of the corresponding quinoline **6j** in 65% yield of the isolated product. Interestingly, when the reaction was conducted in the presence of TBAF (2.5 equiv, condition B), the product was not **6j** but 2-ethoxy-4-dienylquinoline **7a**—the product resulting from the elimination of benzyl alcohol. Quinoline **7a** was obtained in 30% yield via this extended reaction cascade, which terminates with the elimination of benzyl alcohol. Similarly, the reaction of **1b** with 4-methyl-thiazol-5-yl-ethanol (**5c**) in the presence of TBAF yielded 4-dienyl-2-ethoxyquinoline **7b** as the major product in 21% yield.

**Scheme 3:**
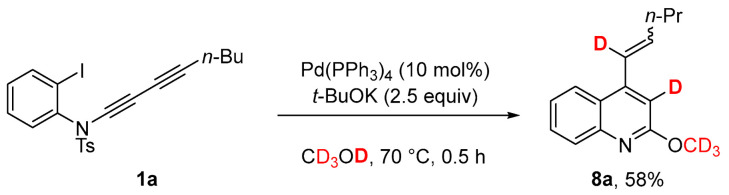
Deuterium labeling experiment.

A deuterium labeling experiment was carried out with diynamide **1a** in CD_3_OD using *t*-BuOK as the base instead of KOH to avoid any proton source (Scheme 3). 

**Scheme 4:**
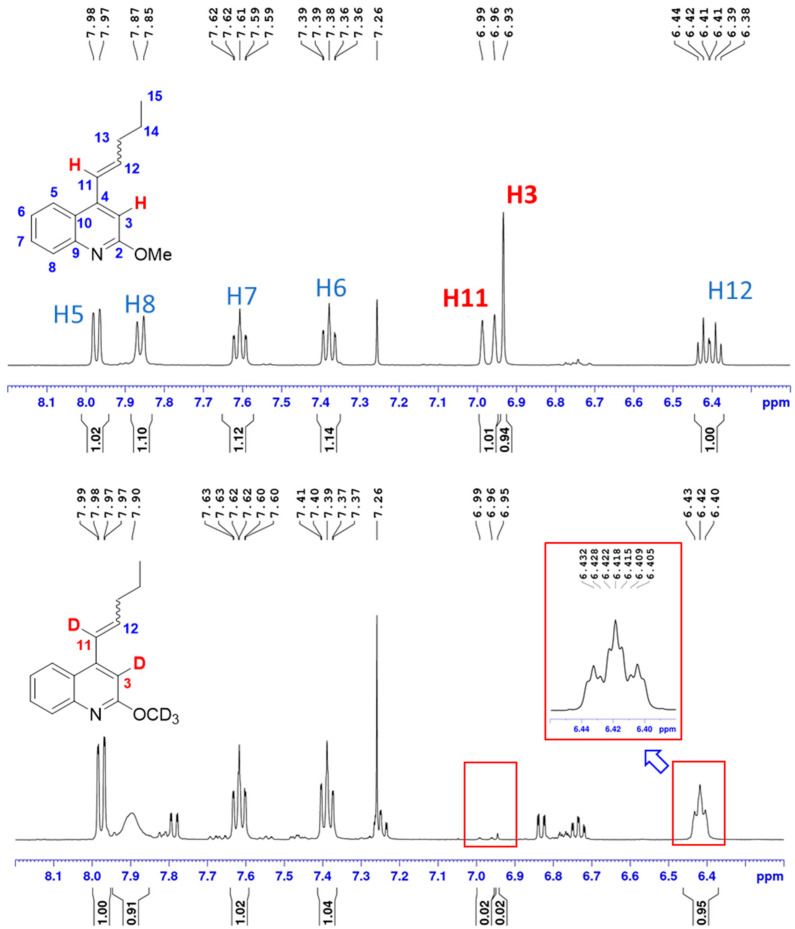
Comparison of the ^1^H NMR (CDCl_3_, 500 MHz) spectra of quinolines **6a** (top) and **8a** (bottom). Only the δ = 6.2–8.2 ppm range is shown for reasons of clarity.

The resulting penta-deuterated quinoline **8a** was isolated in 58% yield. The comparison of the ^1^H NMR spectrum of **8a** with that of non-deuterated quinoline **6a** provided evidence of the selective incorporation of deuterium into the C3 and C11 positions (Scheme 4). The ^1^H NMR signals at δ = 6.93 (s, H-3) and 6.97 (d, H-11) for the non-deuterated quinoline **6a** almost disappeared in the ^1^H NMR spectrum of **8a**. Moreover, the multiplicity of the signal assigned to the olefinic proton H-12 at δ = 6.41 was altered from a doublet of triplets with *^3^J_H,H_* = 15.5 Hz and *^3^J_H,H_* = 7.0 Hz to a triplet of triplets with *^3^J_H,H_* = 7.0 Hz and *^3^J_H,D(trans)_* = 2.0 Hz. The clear loss of the ^1^H signals at δ = 6.93 and 6.97, as well as the clear changes in the spin-system of the proton signal at δ = 6.41 in the ^1^H NMR spectra indicate an almost complete incorporation of deuterium at those positions.

**Scheme 5:**
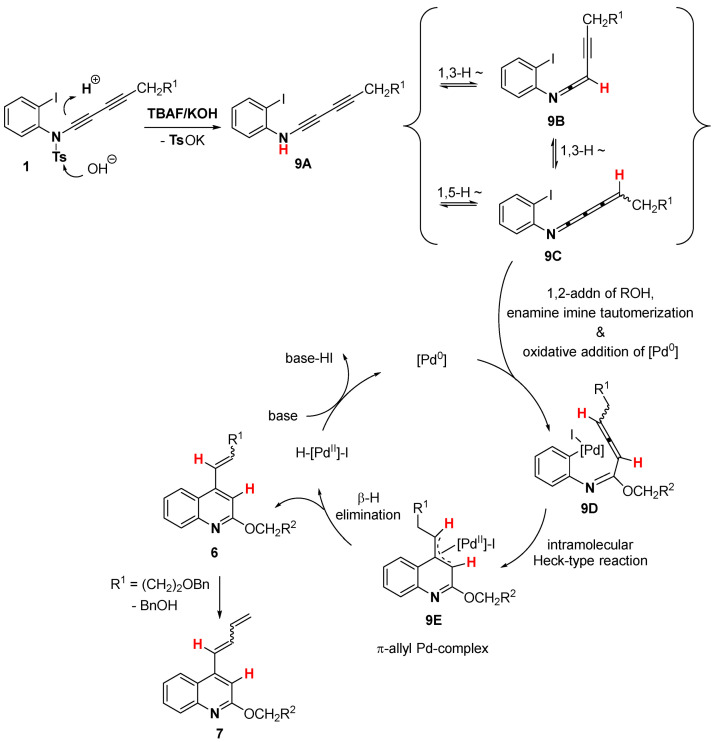
Proposed mechanism for the synthesis of 2-alkoxy-4-alkenylquinolines **6** and 2-alkoxy-4-(1,3-dienyl)quinolines **7**.

The deuterium labelling experiment supports well the proposed mechanism outlined in Scheme 5. Presumably, the combination of alcohol **5**/KOH or **5**/TBAF/KOH cleaves the tosyl group located at the ynamide nitrogen atom (**1** → **9A**) and initiates the subsequent cascade reaction (Scheme 5). The resulting 1,3-butadiynamine **9A** is in equilibrium with the alkynyl ketenimine **9B** and the [4]-cumulenimine **9C** with formal 1,3- and 1,5-H shifts, respectively. Selective 1,2-addition of alcohol **5** to either imine **9B** or **9C** followed by enamine-imine tautomerization—and isomerization of the propargyl to an allene moiety in the case of **9B**—delivers the allene-derived intermediate **9D** after oxidative addition of the palladium(0) catalyst. Whether the oxidative addition of palladium(0) already occurs with intermediates **9A**–**C** and therefore facilitates the isomerization sequence or whether oxidative addition takes place after the 1,3-diynamine-to-allenyl-imine isomerization remains an open question. However, once the σ,π-chelated palladium intermediate **9D** is formed, a subsequent intramolecular Heck-type reaction furnishes the annulated π-allyl palladium species **9E**, which undergoes β-hydrogen elimination to produce 4-alkenyl quinolines **6** along with the palladium–hydride complex. Reduction of the latter by the base allows the regeneration of the Pd(0) active catalyst. Interestingly, in the case of 1,3-butadiynamide **1b** (R^1^ = (CH_2_)_2_OBn), the reaction conditions indicated by B favor in situ elimination of benzyl alcohol and yield 2-alkoxy-4-(1,3-dienyl)quinolines **7** as the major products. Here, the formation of the conjugated 1,3-butadiene moiety is most likely the underlying driving force.

## 3. Materials and Methods

Chromatographic purification steps were performed using Acros Organics silica gel Si 60 (40–60 μm) or Merck aluminum oxide 90 active neutral (60–200 μm, activity stage III). Thin-layer chromatography (TLC) was developed on Merck silica gel 60 plates (0.20 mm) or on aluminum oxide 60 neutral plates (0.20 mm) with UV detection (Merck, Kenilworth, NJ, USA). Nuclear magnetic resonance (NMR) spectra were recorded on a BRUKER AVANCE III 500 (500 MHz) or a BRUKER NEO 600 (600 MHz) spectrometer (Bruker, Billerica, Kenilworth, MA, USA). ^1^H and ^13^C NMR chemical shifts (δ) are given in ppm (parts per million) using the TMS signal (0 ppm) and the residual peak of chloroform-D (77.16 ppm), respectively, as the internal reference (see Supplementary Materials). Coupling constants are reported in Hertz (Hz). The following abbreviations are used: s = singlet, d = doublet, t = triplet, q = quartet, qt = quintuplet, sext = sextuplet, m = multiplet, br = broad). The (*E*)/(*Z*) isomer ratio was determined for each quinoline from the ^1^H NMR spectrum via integration of the dt signals of the olefin proton of both isomers. High-resolution mass spectrometry (HRMS) analysis was performed in electron spray ionization (ESI) mode on an Acquity UPLC H-ClassXevo G2-XS QTof (WATERS) spectrometer (Waters, Milford, MA, USA). Melting points (Mp) were measured with an electrothermal apparatus and are not corrected. The procedure for the synthesis of 1,3-butadiynamides **1a,b** and their characterization data were reported [32,33].

### 3.1. General Procedure for the Synthesis of 2-Alkoxyquinolines ***6a***–***b***, ***f***, ***j*** (Condition A)

1,3-butadiynamide **1** (0.11 mmol), KOH (15 mg, 0.26 mmol, 2.5 equiv), tetrakis(triphenylphosphine)palladium(0) (Pd(PPh_3_)_4_) (12 mg, 10 mol%) and alcohol **5** (1 mL) were introduced in a dry Schlenk tube under an argon atmosphere. The Schlenk tube was immediately placed in an oil bath preheated to 70 °C. After 1 h, CH_2_Cl_2_ and brine were added to the reaction mixture. The aqueous phase was extracted with CH_2_Cl_2_, and the combined organic layers were dried (MgSO_4_), filtered and evaporated under reduced pressure. The product was purified via silica gel or alumina column chromatography.

*2-Methoxy-4-(pent-1-en-1-yl)quinoline* (**6a**). Prepared from *N*-(2-iodophenyl)-4-methyl-*N*-(octa-1,3-diyn-1-yl)benzenesulfonamide (**1a**) (50 mg, 0.105 mmol) and MeOH (**5a**) (1 mL). The product was purified via column chromatography (Al_2_O_3_, *n*-pentane/Et_2_O 20:1 (*v*/*v*)) to yield **6a** as an oil (18 mg, 0.08 mmol, yield: 75%, E/Z = 96:4). R_f_ = 0.53 (*n*-pentane/Et_2_O = 20:1 (*v*/*v*)). ^1^H NMR (500 MHz, CDCl_3_) δ 7.97 (d, *^3^J* = 7.9 Hz, 1 H), 7.86 (d, *^3^J* = 8.3 Hz, 1 H), 7.61 (ddd, *^3^J* = 8.3 Hz, *^3^J* = 7.0 Hz, *^4^J* = 1.2 Hz, 1 H), 7.38 (ddd, *^3^J* = 8.1 Hz, *^3^J* = 7.0 Hz, *^4^J* = 1.3 Hz, 1 H), 6.97 (d, *^3^J* = 15.7 Hz, 1 H), 6.93 (s, 1 H), 6.41 (dt, *^3^J* = 15.5 Hz, *^3^J* = 7.0 Hz, 1 H), 4.07 (s, 3 H), 2.31 (m, 2 H), 1.57 (sext, *^3^J* = 7.4 Hz, 2 H) and 1.00 (t, *^3^J* = 7.4 Hz, 3 H). Identified ^1^H NMR signals corresponding to the minor isomer: 6.02 (dt, *^3^J* = 11.6 Hz, *^3^J* = 7.5 Hz, 1 H). ^13^C NMR (125 MHz, CDCl_3_) δ 162.7 (C), 147.1 (C), 146.7 (C), 138.0 (CH), 129.4 (CH), 127.8 (CH), 124.9 (CH), 123.89 (CH), 123.85 (C), 123.8 (CH), 108.7 (CH), 53.4 (CH_3_), 35.6 (CH_2_), 22.4 (CH_2_) and 13.9 (CH_3_). HRMS (ESI+): Calc’d for C_15_H_18_NO [M + H]^+^: 228.1388; found: 228.1395.

*2-Ethoxy-4-(pent-1-en-1-yl)quinoline* (**6b**). Prepared from **1a** (50 mg, 0.105 mmol) in EtOH (**5b**) (1 mL). The product was purified via column chromatography (Al_2_O_3_, *n*-pentane/Et_2_O 20:1 (*v*/*v*)) to yield **6b** as an oil (20 mg, 0.083 mmol, yield: 79%, E/Z = 97:3). R_f_ = 0.62 (*n*-pentane/Et_2_O = 20:1 (*v*/*v*)). ^1^H NMR (600 MHz, CDCl_3_) δ 7.96 (dd, *^3^J* = 8.3 Hz, *^4^J* = 1.1 Hz, 1 H), 7.83 (d, *^3^J* = 8.3 Hz, 1 H), 7.59 (ddd, *^3^J* = 8.3 Hz, *^3^J* = 6.9 Hz, *^4^J* = 1.4 Hz, 1 H), 7.36 (ddd, *^3^J* = 8.2 Hz, *^3^J* = 7.0 Hz, *^4^J* = 1.3 Hz, 1 H), 6.97 (d, *^3^J* = 15.6 Hz, 1 H), 6.93 (s, 1 H), 6.41 (dt, *^3^J* = 15.5 Hz, *^3^J* = 7.0 Hz, 1 H), 4.53 (q, *^3^J* = 7.1 Hz, 2 H), 2.30 (m, 2 H), 1.57 (sext, *^3^J* = 7.4 Hz, 2 H), 1.45 (t, *^3^J* = 7.1 Hz, 3 H) and 1.00 (t, *^3^J* = 7.4 Hz, 3 H). Identified ^1^H NMR signals corresponding to the minor isomer: 6.01 (dt, *^3^J* = 11.6 Hz, *^3^J* = 7.5 Hz, 1 H). ^13^C NMR (150 MHz, CDCl_3_) δ 162.4 (C), 147.2 (C), 146.6 (C), 137.8 (CH), 129.4 (CH), 127.8 (CH), 124.9 (CH), 123.78 (C), 123.76 (CH), 123.7 (CH), 108.9 (CH), 61.7 (CH_2_), 35.6 (CH_2_), 22.4 (CH_2_), 14.8 (CH_3_) and 13.9 (CH_3_). HRMS (ESI+): Calc’d for C_16_H_20_NO [M + H]^+^: 242.1545; found: 242.1547.

*N,N-Dimethyl-2-((4-(pent-1-en-1-yl)quinolin-2-yl)oxy)ethanamine* (**6f**). Prepared from **1a** (50 mg, 0.105 mmol) and 3-(dimethylamino)propan-1-ol (**5f**) (105 μL, 1.1 mmol, 10 equiv) in dry THF (1 mL). The reaction was complete after 30 min at 70 °C. The product was purified via column chromatography (Al_2_O_3_, *n*-pentane/EtOAc/Et_3_N 90:10:1 (*v*/*v*/*v*)) to yield **6f** (19 mg, 0.067 mmol, yield: 64%, *E*/*Z* = 97:3) as an oil. R_f_ = 0.37 (*n*-pentane/EtOAc/Et_3_N 90:10:1 (*v*/*v*/*v*)). ^1^H NMR (500 MHz, CDCl_3_) δ 7.97 (dd, *^3^J* = 8.3 Hz, *^4^J* = 1.0 Hz, 1 H), 7.82 (d, *^3^J* = 8.3 Hz, 1 H), 7.59 (ddd, *^3^J* = 8.4 Hz, *^3^J* = 7.0 Hz, *^4^J* = 1.4 Hz, 1 H), 7.37 (ddd, *^3^J* = 8.2 Hz, *^3^J* = 7.0 Hz, *^4^J* = 1.2 Hz, 1 H), 7.01 (s, 1 H), 6.97 (d, *^3^J* = 15.6 Hz, 1 H), 6.40 (dt, *^3^J* = 15.6 Hz, *^3^J* = 7.0 Hz, 1 H), 4.59 (t, *^3^J* = 5.6 Hz, 2 H), 2.78 (t, *^3^J* = 5.6 Hz, 2 H), 2.37 (s, 6 H), 2.30 (m, 2 H), 1.56 (sext, *^3^J* = 7.3 Hz, 2 H) and 1.00 (t, *^3^J* = 7.3 Hz, 3 H). Identified ^1^H NMR signals corresponding to the minor isomer: 6.01 (dt, *^3^J* = 11.6 Hz, *^3^J* = 7.4 Hz, 1 H). ^13^C NMR (125 MHz, CDCl_3_) δ 162.3 (C), 147.1 (C), 146.5 (C), 137.9 (CH), 129.3 (CH), 127.8 (CH), 124.8 (CH), 123.9 (C), 123.8 (CH), 123.7 (CH), 109.1 (CH), 63.2 (CH_2_), 58.4 (CH_2_), 45.9 (CH_3_), 35.5 (CH_2_), 22.4 (CH_2_) and 13.8 (CH_3_). HRMS (ESI+): Calc’d for C_18_H_25_N_2_O [M + H]^+^: 285.1967; found: 285.1968.

*(E)-4-(4-(Benzyloxy)but-1-en-1-yl)-2-ethoxyquinoline* (**6j**). Prepared from *N*-(7-(benzyloxy)hepta-1,3-diyn-1-yl)-*N*-(2-iodophenyl)-4-methylbenzenesulfonamide (**1b**) (50 mg, 0.088 mmol) in EtOH (1 mL). The reaction was complete after 30 min at 70 °C. The product was purified via column chromatography (SiO_2_, *n*-pentane/Et_2_O 20:1 (*v*/*v*)) to yield (*E*)-**6j** as an oil (19 mg, 0.057 mmol, yield: 65%). R_f_ = 0.25 (*n*-pentane/Et_2_O 20:1 (*v*/*v*)). ^1^H NMR (600 MHz, CDCl_3_) δ 7.94 (d, *^3^J* = 8.2 Hz, 1 H), 7.82 (d, *^3^J* = 8.3 Hz, 1 H), 7.60 (dd, *^3^J* = 8.0 Hz, *^3^J* = 7.1 Hz, 1 H), 7.36-7.28 (m, 6 H), 7.05 (d, *^3^J* = 15.7 Hz, 1 H), 6.93 (s, 1 H), 6.43 (dt, *^3^J* = 15.6 Hz, *^3^J* = 7.0 Hz, 1 H), 4.57 (s, 2 H), 4.52 (q, *^3^J* = 7.1 Hz, 2 H), 3.66 (t, *^3^J* = 6.5 Hz, 2 H), 2.65 (m, 2 H) and 1.45 (t, *^3^J* = 7.1 Hz, 3 H). ^13^C NMR (150 MHz, CDCl_3_) δ 162.4 (C), 147.2 (C), 146.2 (C), 138.4 (C), 134.0 (CH), 129.4 (CH), 128.6 (CH), 127.9 (CH), 127.82 (CH), 127.80 (CH), 126.6 (CH), 123.8 (CH), 123.72 (CH), 123.69 (C), 109.1 (CH), 73.2 (CH_2_), 69.5 (CH_2_), 61.6 (CH_2_), 34.0 (CH_2_) and 14.8 (CH_3_). HRMS (ESI+): Calc’d for C_22_H_24_NO_2_ [M + H]^+^: 334.1807; found: 334.1808.

### 3.2. General Procedure for the Synthesis of 2-Alkoxyquinolines ***6c***–***e***, ***g***-***i*** and ***7a***–***b*** (Condition B)

1,3-butadiynamide **1** (0.11 mmol), KOH (15 mg, 0.26 mmol, 2.5 equiv), Pd(PPh_3_)_4_ (12 mg, 10 mol%), alcohol **5** (10–20 equiv), tetra-*n*-butylammonium fluoride (TBAF) 1M solution in THF (0.3 mL, 0.3 mmol, 2.5 equiv) and dry THF (1 mL) were introduced in a Schlenk tube under an argon atmosphere. The Schlenk tube was immediately placed in an oil bath preheated to 70 °C. Upon completion of the reaction (0.5 to 1 h), CH_2_Cl_2_ and brine were added to the reaction mixture. The aqueous phase was extracted with CH_2_Cl_2_ and the combined organic layers were dried (MgSO_4_), filtered and evaporated under reduced pressure. The product was purified via silica gel or alumina column chromatography.

*4-Methyl-5-(2-((4-(pent-1-en-1-yl)quinolin-2-yl)oxy)ethyl)thiazole* (**6c**). Prepared from **1a** (50 mg, 0.105 mmol) and 2-(4-methylthiazol-5-yl)ethanol (**5c**) (0.25 mL, 2.1 mmol, 20 equiv) in 1 mL of 1*M* tetra-*n*-butylammonium fluoride (TBAF) solution in THF (1 mmol, 9.5 equiv). The reaction was complete after 1 h at 70 °C. The product was purified via column chromatography (SiO_2_, *n*-pentane/EtOAc/Et_3_N 90:10:1 (*v*/*v*/*v*)) to yield **6c** as an oil (14 mg, 0.041 mmol, yield: 39%, *E*/*Z* = 95:5). R_f_ = 0.20 (*n*-pentane/EtOAc/Et_3_N 90:10:1 (*v*/*v*/*v*)). ^1^H NMR (600 MHz, CDCl_3_) δ 8.59 (s, 1 H), 7.98 (dd, *^3^J* = 8.3 Hz, *^4^J* = 1.0 Hz, 1 H), 7.82 (d, *^3^J* = 8.3 Hz, 1 H), 7.61 (ddd, *^3^J* = 8.3 Hz, *^3^J* = 7.0 Hz, *^4^J* = 1.4 Hz, 1 H), 7.38 (ddd, *^3^J* = 8.2 Hz, *^3^J* = 7.0 Hz, *^4^J* = 1.3 Hz, 1 H), 6.98 (d, *^3^J* = 15.8 Hz, 1 H), 6.93 (s, 1 H), 6.42 (dt, *^3^J* = 15.6 Hz, *^3^J* = 7.0 Hz, 1 H), 4.64 (t, *^3^J* = 6.7 Hz, 2 H), 3.30 (t, *^3^J* = 6.7 Hz, 2 H), 2.48 (s, 3 H), 2.32 (m, 2 H), 1.58 (sext, *^3^J* = 7.3 Hz, 2 H) and 1.01 (t, *^3^J* = 7.4 Hz, 3 H). Identified ^1^H NMR signals corresponding to the minor isomer: 6.03 (dt, *^3^J* = 11.6 Hz, *^3^J* = 7.5 Hz, 1 H). ^13^C NMR (150 MHz, CDCl_3_) δ 161.8 (C), 149.9 (C), 149.8 (CH), 147.0 (C), 146.9 (C), 138.1 (CH), 129.5 (CH), 127.8 (CH), 127.7 (C), 124.8 (CH), 124.0 (CH), 123.9 (C), 123.8 (CH), 108.6 (CH), 65.4 (CH_2_), 35.6 (CH_2_), 26.3 (CH_2_), 22.4 (CH_2_), 15.2 (CH_3_) and 13.9 (CH_3_). HRMS (ESI+): Calc’d for C_20_H_23_N_2_OS [M + H]^+^: 339.1531; found: 339.1530.

*2-((4-(pent-1-en-1-yl)quinolin-2-yl)oxy)ethanol* (**6d**). Prepared from **1a** (50 mg, 0.105 mmol) and ethylene glycol (**5d**) (120 μL, 2.1 mmol, 20 equiv). The reaction was complete after 1 h at 70 °C. The product was purified via column chromatography (SiO_2_, *n*-pentane/EtOAc/Et_3_N 80:20:1 (*v*/*v*/*v*) to 60:40:1 (*v*/*v*/*v*)) to yield **6d** as an oil (12 mg, 0.047 mmol, yield: 45%, *E*/*Z* = 98:2). R_f_ = 0.17 (pentane/EtOAc/Et_3_N 80:20:1 (*v*/*v*/*v*)). ^1^H NMR (600 MHz, CDCl_3_) δ 7.98 (d, *^3^J* = 8.2 Hz, 1 H), 7.80 (d, *^3^J* = 8.2 Hz, 1 H), 7.62 (ddd, *^3^J* = 8.3 Hz, *^3^J* = 7.0 Hz, *^4^J* = 1.3 Hz, 1 H), 7.40 (ddd, *^3^J* = 8.2 Hz, *^3^J* = 7.0 Hz, *^4^J* = 1.1 Hz, 1 H), 6.99 (s, 1 H), 6.98 (d, *^3^J* = 15.4 Hz, 1 H), 6.43 (dt, *^3^J* = 15.4 Hz, *^3^J* = 7.0 Hz, 1 H), 4.74 (br s, 1 H), 4.64 (m, 2 H), 4.00 (m, 2 H), 2.32 (m, 2 H), 1.57 (sext, *^3^J* = 7.4 Hz, 2 H) and 1.00 (t, *^3^J* = 7.4 Hz, 3 H). Identified ^1^H NMR signals corresponding to the minor isomer: 6.05 (dt, *^3^J* = 11.6 Hz, *^3^J* = 7.4 Hz, 1 H). ^13^C NMR (150 MHz, CDCl_3_) δ 162.6 (C), 147.5 (C), 146.3 (C), 138.5 (CH), 129.8 (CH), 127.3 (CH), 124.7 (CH), 124.3 (CH), 123.9 (C), 123.8 (CH), 108.8 (CH), 69.6 (CH_2_), 63.1 (CH_2_), 35.6 (CH_2_), 22.3 (CH_2_) and 13.9 (CH_3_). HRMS (ESI+): Calc’d for C_16_H_20_NO_2_ [M + H]^+^: 258.1494; found: 258.1497.

*4-((4-(pent-1-en-1-yl)quinolin-2-yl)oxy)butan-2-ol* (**6e**). Prepared from **1a** (50 mg, 0.105 mmol) and 1,3-butanediol (**5e**) (190 μL, 2.1 mmol, 20 equiv). The reaction was complete after 40 min at 70 °C. The product was purified via column chromatography (SiO_2_, *n*-pentane/AcOEt/Et_3_N 90:10:1 (*v*/*v*/*v*) to 80:20:1 (*v*/*v*/*v*)) to yield **6e** as an oil (19 mg, 0.067 mmol, yield: 63%, *E*/*Z* = 98:2). R_f_ = 0.65 (*n*-pentane/AcOEt 6:4 (*v*/*v*)). ^1^H NMR (600 MHz, CDCl_3_) δ 7.98 (dd, *^3^J* = 8.3 Hz, *^4^J* = 1.1 Hz, 1 H), 7.80 (d, *^3^J* = 8.2 Hz, 1 H), 7.61 (ddd, *^3^J* = 8.3 Hz, *^3^J* = 7.0 Hz, *^4^J* = 1.4 Hz, 1 H), 7.39 (ddd, *^3^J* = 8.2 Hz, *^3^J* = 7.0 Hz, *^4^J* = 1.3 Hz, 1 H), 6.97 (d, *^3^J* = 15.6 Hz, 1 H), 6.93 (s, 1 H), 6.43 (dt, *^3^J* = 15.5 Hz, *^3^J* = 7.0 Hz, 1 H), 5.09 (m, 1 H), 4.79 (br s, 1 H), 4.34 (m, 1 H), 3.83 (m, 1 H), 2.32 (m, 2 H), 2.01 (m, 1 H), 1.76 (m, 1 H), 1.57 (sext, *^3^J* = 7.4 Hz, 2 H), 1.23 (d, *^3^J* = 6.2 Hz, 3 H) and 1.00 (t, *^3^J* = 7.4 Hz, 3 H). Identified ^1^H NMR signals corresponding to the minor isomer: 6.03 (dt, *^3^J* = 11.7 Hz, *^3^J* = 7.4 Hz, 1 H). ^13^C NMR (150 MHz, CDCl_3_) δ 162.8 (C), 147.4 (C), 146.3 (C), 138.5 (CH), 129.9 (CH), 127.2 (CH), 124.7 (CH), 124.2 (CH), 123.79 (C), 123.78 (CH), 108.8 (CH), 63.7 (CH), 63.0 (CH_2_), 39.9 (CH_2_), 35.6 (CH_2_), 22.9 (CH_3_), 22.3 (CH_2_) and 13.9 (CH_3_). HRMS (ESI+): Calc’d for C_18_H_24_NO_2_ [M + H]^+^: 286.1807; found: 286.1806.

*2-((3-Methylbut-3-en-1-yl)oxy)-4-(pent-1-en-1-yl)quinoline* (**6g**). Prepared from **1a** (50 mg, 0.105 mmol) and 2-methylprop-2-en-1-ol (**5g**) (215 μL, 2.1 mmol, 20 equiv). The reaction was complete after 45 min at 70 °C. The product was purified via column chromatography (SiO_2_, *n*-pentane/Et_2_O 20:1 (*v*/*v*)) to yield **6g** as an oil (22 mg, 0.078 mmol, yield: 75%, *E*/*Z* = 97:3). R_f_ = 0.65 (*n*-pentane/Et_2_O 20:1 (*v*/*v*)). ^1^H NMR (600 MHz, CDCl_3_) δ 7.97 (dd, *^3^J* = 8.3 Hz, *^4^J* = 1.0 Hz, 1 H), 7.84 (dd, *^3^J* = 8.3 Hz, *^4^J* = 0.8 Hz, 1 H), 7.60 (ddd, *^3^J* = 8.3 Hz, *^3^J* = 6.8 Hz, *^4^J* = 1.4 Hz, 1 H), 7.37 (ddd, *^3^J* = 8.2 Hz, *^3^J* = 7.0 Hz, *^4^J* = 1.3 Hz, 1 H), 6.97 (d, *^3^J* = 15.8 Hz, 1 H), 6.94 (s, 1 H), 6.42 (dt, *^3^J* = 15.5 Hz, *^3^J* = 7.0 Hz, 1 H), 4.85 (s, 2 H), 4.59 (t, *^3^J* = 6.8 Hz, 2 H), 2.55 (t, *^3^J* = 6.8 Hz, 2 H), 2.30 (m, 2 H), 1.85 (s, 3 H), 1.56 (sext, *^3^J* = 7.4 Hz, 2 H) and 1.00 (t, *^3^J* = 7.4 Hz, 3 H). Identified ^1^H NMR signals corresponding to the minor isomer: 6.01 (dt, *^3^J* = 11.6 Hz, *^3^J* = 7.4 Hz, 1 H). ^13^C NMR (150 MHz, CDCl_3_) δ 162.4 (C), 147.2 (C), 146.6 (C), 142.7 (C), 137.8 (CH), 129.3 (CH), 127.8 (CH), 124.9 (CH), 123.83 (C), 123.78 (CH), 123.7 (CH), 112.0 (CH_2_), 108.9 (CH), 64.1 (CH_2_), 37.3 (CH_2_), 35.6 (CH_2_), 22.9 (CH_3_), 22.4 (CH_2_) and 13.9 (CH_3_). HRMS (ESI+): Calc’d for C_19_H_24_NO [M + H]^+^: 282.1858; found: 282.1863.

*(E)-2-(Benzo[d][1,3]dioxol-5-ylmethoxy)-4-(pent-1-en-1-yl)quinoline* (**6h**). Prepared from **1a** (50 mg, 0.105 mmol) and benzo[d][1,3]dioxol-5-ylmethanol (**5h**) (320 mg, 2.1 mmol, 20 equiv). The reaction was complete after 30 min at 70 °C. The product was purified via column chromatography (SiO_2_, *n*-pentane/Et_2_O 20:1 (*v*/*v*)) to yield (*E*)-**6h** as an oil (21 mg, 0.06 mmol, yield: 58%). R_f_ = 0.48 (*n*-pentane/Et_2_O 20:1 (*v*/*v*)). ^1^H NMR (600 MHz, CDCl_3_) δ 7.98 (dd, *^3^J* = 8.3 Hz, *^4^J* = 1.1 Hz, 1 H), 7.86 (dd, *^3^J* = 8.3 Hz, *^4^J* = 1.0 Hz, 1 H), 7.61 (ddd, *^3^J* = 8.3 Hz, *^3^J* = 6.9 Hz, *^4^J* = 1.4 Hz, 1 H), 7.39 (ddd, *^3^J* = 8.3 Hz, *^3^J* = 6.9 Hz, *^4^J* = 1.3 Hz, 1 H), 7.04 (d, *^4^J* = 1.6 Hz, 1 H), 7.00 (dd, *^3^J* = 7.9 Hz, *^4^J* = 1.6 Hz, 1 H), 6.98 (s, 1 H), 6.97 (d, *^3^J* = 15.6 Hz, 1 H), 6.82 (d, *^3^J* = 7.9 Hz, 1 H), 6.40 (dt, *^3^J* = 15.6 Hz, *^3^J* = 7.0 Hz, 1 H), 5.96 (s, 2 H), 5.44 (s, 2 H), 2.30 (m, 2 H), 1.56 (sext, *^3^J* = 7.4 Hz, 2 H) and 0.99 (t, *^3^J* = 7.4 Hz, 3 H). ^13^C NMR (150 MHz, CDCl_3_) δ 162.0 (C), 147.8 (C), 147.4 (C), 147.1 (C), 146.8 (C), 138.0 (CH), 131.3 (C), 129.4 (CH), 127.9 (CH), 124.8 (CH), 124.0 (CH), 123.9 (C), 123.7 (CH), 122.3 (CH), 109.3 (CH), 108.9 (CH), 108.3 (CH), 101.2 (CH_2_), 67.6 (CH_2_), 35.6 (CH_2_), 22.4 (CH_2_) and 13.9 (CH_3_). HRMS (ESI+): Calc’d for C_22_H_22_NO_3_ [M + H]^+^: 348.1600; found: 348.1601.

*2-(4-Fluorophenyl)ethoxy)-4-(pent-1-en-1-yl)quinoline* (**6i**). Prepared from **1a** (50 mg, 0.105 mmol) and 2-(4-fluorophenyl)ethanol (**5i**) (130 μL, 1.05 mmol, 10 equiv). The reaction was complete after 40 min at 70 °C. The product was purified via column chromatography (SiO_2_, *n*-pentane/Et_2_O 98:2 (*v*/*v*)) to yield **6i** as a white solid (14 mg, 0.04 mmol, yield: 40%, *E*/*Z* = 98:2). Mp 48–49 °C. R_f_ = 0.53 (*n*-pentane/EtOAc 95:5 (*v*/*v*)). ^1^H NMR (600 MHz, CDCl_3_) δ 7.96 (d, *^3^J* = 8.2 Hz, 1 H), 7.81 (d, *^3^J* = 8.4 Hz, 1 H), 7.59 (ddd, *^3^J* = 8.2 Hz, *^3^J* = 7.0 Hz, *^4^J* = 1.3 Hz, 1 H), 7.37 (ddd, *^3^J* = 8.2 Hz, *^3^J* = 7.0 Hz, *^4^J* = 1.1 Hz, 1 H), 7.29-7.27 (m, 2 H), 7.00 (m, 2 H), 6.96 (d, *^3^J* = 15.6 Hz, 1 H), 6.91 (s, 1 H), 6.40 (dt, *^3^J* = 15.6 Hz, *^3^J* = 7.0 Hz, 1 H), 4.66 (t, *^3^J* = 7.0 Hz, 2 H), 3.11 (t, *^3^J* = 7.0 Hz, 2 H), 2.30 (m, 2 H), 1.56 (sext, *^3^J* = 7.4 Hz, 2 H) and 1.00 (t, *^3^J* = 7.4 Hz, 3 H). Identified ^1^H NMR signals corresponding to the minor isomer: 6.02 (dt, *^3^J* = 11.7 Hz, *^3^J* = 7.4 Hz, 1 H). ^13^C NMR (150 MHz, CDCl_3_) δ 161.7 (d, *^1^J_C-F_* = 242 Hz, C), 162.2 (C), 147.2 (C), 146.7 (C), 137.9 (CH), 134.5 (d, *^4^J_C-F_* = 3 Hz, C), 130.6 (d, *^3^J_C-F_* = 8 Hz, CH), 129.4 (CH), 127.8 (CH), 124.8 (CH), 123.9 (CH), 123.8 (C), 123.7 (CH), 115.3 (d, *^2^J_C-F_* = 21 Hz, CH), 108.8 (CH), 66.3 (CH_2_), 35.6 (CH_2_), 34.8 (CH_2_), 22.4 (CH_2_) and 13.9 (CH_3_). ^19^F{H} NMR (470 MHz, CDCl_3_) δ -117.0. HRMS (ESI+): Calc’d for C_22_H_23_NOF [M + H]^+^: 336.1764; found: 336.1761.

*(E)-2-Ethoxy-4-(buta-1,3-dien-1-yl)quinoline* (**7a**). Prepared from *N*-(7-(benzyloxy)hepta-1,3-diyn-1-yl)-*N*-(2-iodophenyl)-4-methylbenzenesulfonamide (**1b)** (57 mg, 0.1 mmol) and EtOH (**5b**) (120 μL, 2.0 mmol, 20 equiv). The reaction was complete after 50 min at 70 °C. The product was purified via column chromatography (SiO_2_, *n*-pentane/EtOAc 98:2 (*v*/*v*)) to yield (*E*)-**7a** as a white solid (7 mg, 0.03 mmol, yield: 30%). Mp 64–65 °C. R_f_ = 0.4 (*n*-pentane/EtOAc 98:2 (*v*/*v*)). ^1^H NMR (600 MHz, CDCl_3_) δ 7.97 (dd, *^3^J* = 8.3 Hz, *^4^J* = 1.1 Hz, 1 H), 7.83 (dd, *^3^J* = 8.3 Hz, *^4^J* = 0.7 Hz, 1 H), 7.61 (ddd, *^3^J* = 8.3 Hz, *^3^J* = 6.9 Hz, *^4^J* = 1.4 Hz, 1 H), 7.38 (ddd, *^3^J* = 8.3 Hz, *^3^J* = 6.9 Hz, *^4^J* = 1.3 Hz, 1 H), 7.17 (br d, *^3^J* = 15.4 Hz, 1 H), 7.01 (s, 1 H), 6.95 (ddt, *^3^J* = 15.4 Hz, *^3^J* = 10.5 Hz, *^4^J* = 0.7 Hz, 1 H), 6.64 (dddd, *^3^J* = 16.9 Hz, *^3^J* = 10.6 Hz, *^3^J* = 10.0 Hz, *^4^J* = 0.7 Hz, 1 H), 5.47 (dddd, *^3^J* = 16.9 Hz, *^2^J* = 1.4 Hz, *^4^J* = 0.7 Hz, *^5^J* = 0.7 Hz, 1 H), 5.34 (dddd, *^3^J* = 10.0 Hz, *^2^J* = 1.4 Hz, *^4^J* = 0.7 Hz, *^5^J* = 0.7 Hz, 1 H), 4.53 (q, *^3^J* = 7.1 Hz, 2 H) and 1.45 (t, *^3^J* = 7.1 Hz, 3 H). ^13^C NMR (150 MHz, CDCl_3_) δ 162.3 (C), 147.4 (C), 145.3 (C), 136.8 (CH), 135.5 (CH), 129.5 (CH), 128.0 (CH), 127.0 (CH), 123.9 (CH), 123.6 (C), 123.4 (CH), 120.6 (CH_2_), 108.7 (CH), 61.7 (CH_2_) and 14.8 (CH_3_). HRMS (ESI+): Calc’d for C_15_H_16_NO [M + H]^+^: 226.1232; found: 226.1233.

*(E)-5-(2-((4-(buta-1,3-dien-1-yl)quinolin-2-yl)oxy)ethyl)-4-methylthiazole* (**7b**). Prepared from **1b** (50 mg, 0.088 mmol) and 2-(4-methylthiazol-5-yl)ethanol (**5c**) (210 μL, 1.8 mmol, 20 equiv). The reaction was complete after 50 min at 70 °C. The product was purified via column chromatography (SiO_2_, *n*-pentane/EtOAc/Et_3_N 90:10:1 (*v*/*v*/*v*)) to yield (*E*)-**7b** (6 mg, 0.019 mmol, yield: 21%) as an oil. R_f_ = 0.19 (*n*-pentane/EtOAc 90:10 (*v*/*v*)). ^1^H NMR (600 MHz, CDCl_3_) δ 8.60 (s, 1 H), 7.99 (dd, *^3^J* = 8.3 Hz, *^4^J* = 0.9 Hz, 1 H), 7.83 (dd, *^3^J* = 8.3 Hz, *^4^J* = 0.7 Hz, 1 H), 7.62 (ddd, *^3^J* = 8.3 Hz, *^3^J* = 6.9 Hz, *^4^J* = 1.3 Hz, 1 H), 7.41 (ddd, *^3^J* = 8.2 Hz, *^3^J* = 7.0 Hz, *^4^J* = 1.2 Hz, 1 H), 7.18 (d, *^3^J* = 15.5 Hz, 1 H), 7.02 (s, 1 H), 6.96 (dd, *^3^J* = 15.4 Hz, *^3^J* = 10.6 Hz, 1 H), 6.65 (ddd, *^3^J* = 16.9 Hz, *^3^J* = 10.4 Hz, *^3^J* = 10.1 Hz, 1 H), 5.50 (d, *^3^J* = 16.9 Hz, 1 H), 5.36 (d, *^3^J* = 10.0 Hz, 1 H), 4.65 (t, *^3^J* = 6.7 Hz, 2 H), 3.31 (t, *^3^J* = 6.7 Hz, 2 H) and 2.49 (s, 3 H). ^13^C NMR (150 MHz, CDCl_3_) δ 161.8 (C), 149.94 (C), 149.88 (CH), 147.2 (C), 145.6 (C), 136.8 (CH), 135.7 (CH), 129.7 (CH), 128.0 (CH), 127.7 (C), 126.8 (CH), 124.2 (CH), 123.7 (C), 123.5 (CH), 120.8 (CH_2_), 108.4 (CH), 65.5 (CH_2_), 26.3 (CH_2_) and 15.2 (CH_3_). HRMS (ESI+): Calc’d for C_19_H_19_N_2_OS [M + H]^+^: 323.1218; found: 323.1218.

### 3.3. Deuterium Labeling Experiments

1,3-butadiynamide **1a** (48 mg, 0.10 mmol), tetrakis(triphenylphosphine)palladium(0) (12 mg, 0.01 mmol, 10 mol%) and *t*-BuOK (28 mg, 0.25 mmol, 2.5 equiv) were introduced in a dry Schlenk tube under argon atmosphere. Then CD_3_OD (0.75 mL) was added. The Schlenk tube was sealed and immediately placed in an oil bath preheated to 70 °C. After 30 min, the reaction mixture was diluted with EtOAc and washed with brine. After drying (MgSO_4_), the organic layer was filtered and evaporated under reduced pressure. The product was purified via column chromatography (SiO_2_ pretreated with *n*-pentane/Et_2_O/Et_3_N 94:2:2 (*v*/*v*/*v*), *n*-pentane/Et_2_O 98:2 to 95:5 (*v*/*v*)) to yield **8a** as an oil (13.5 mg, 0.058 mmol, yield: 58%). R_f_ = 0.72 (*n*-pentane/Et_2_O 10:1 (*v*/*v*)).

*D_5_-2-Methoxy-4-(pent-1-en-1-yl)quinoline* (**8a**). ^1^H NMR (500 MHz, CDCl_3_) δ 7.98 (dd, *^3^J* = 8.3 Hz, *^4^J* = 1.0 Hz, 1 H, H_5_), 7.90 (m, 1 H, H_8_), 7.62 (ddd, *^3^J* = 8.3 Hz, *^3^J* = 7.0 Hz, *^4^J* = 1.2 Hz, 1 H, H_7_), 7.39 (ddd, *^3^J* = 8.1 Hz, *^3^J* = 7.0 Hz, *^4^J* = 1.0 Hz, 1 H, H_6_), 6.42 (tt, *^3^J* = 7.0 Hz, *^3^J* = 2 Hz, 1 H, H_12_), 2.32 (q*_app_*, *^3^J* = 7.2 Hz, 2 H, H_13_), 1.57 (sext, *^3^J* = 7.4 Hz, 2 H, H_14_) and 1.00 (t, *^3^J* = 7.4 Hz, 3 H, H_15_). HRMS (ESI+): Calc’d for C_15_H_13_D_5_NO [M + H]^+^: 233.1702; found: 233.1704.

## 4. Conclusions

In conclusion, we have developed a straightforward Pd-catalyzed route to 2-alkoxy 4-alkenylquinolines starting from readily accessible 1,3-butadiynamides. Classic synthetic methods for 2-alkoxyquinolines rely on modifications of the quinoline core and usually yield quinolines with simple alkoxy moieties at the 2-position. By contrast, the presented synthesis strategy is based on the de novo assembly of the 2-alkoxyquinoline core from acyclic precursors—1,3-butadiynamides and primary alcohols as external nucleophiles—to access a highly varied set of 2-alkoxyquinoline derivatives that are otherwise difficult to obtain. The removal of the ynamide tosyl group occurs under defined reaction conditions, which include the use of TBAF/KOH in THF. This initiates the in situ generation of highly reactive and sensitive ketenimine and/or [4]cumulenimine species, which trigger a palladium-catalyzed cascade reaction involving bond formation and cleavage as well as several tautomeric isomerization steps, ultimately leading to highly functionalized 2-alkoxyquinolines.

## Data Availability

The data presented in this study are available on request from the authors.

## Supplementary Materials

The following supporting information can be downloaded at: https://www.mdpi.com/article/10.3390/molecules29153505/s1, Experimental procedures and characterization data of 1,3-butadiynamides **1a,b** [32,33] and NMR spectra for all new compounds.
