# Supplementary material for: Pallado-Catalyzed Cascade Synthesis of 2-Alkoxyquinolines from 1,3-Butadiynamides
Source: Molecules. 2024 Jul 26;29(15):3505. doi: 10.3390/molecules29153505 (PMC11314358; doi:10.3390/molecules29153505)
Supplement: Supplementary file 1 [file molecules-29-03505-s001.zip › molecules-3099515-supplementary.pdf]

Supplementary Materials

Pallado-Catalyzed Cascade Synthesis of 2-Alkoxyquinolines from 1,3-Butadiynamides

Illia Lenko, Alexander Mamontov, Carole Alayrac,* and Bernhard Witulski,*

Laboratoire de Chimie Moléculaire et Thio-organique, CNRS UMR 6507, ENSICAEN, Université de Caen, Normandie Univ, 6 BVD Maréchal Juin, 14050 Caen (France)

E-mail: Carole.witulski-alayrac@ensicaen.fr

E-mail: Bernhard.witulski@ensicaen.fr

Content **Page**

1. Synthesis of 1,3-butadiynamides **1a,b** 1
2. Optimization Table of the reaction conditions of quinoline synthesis 3
3. Comparison of ^{1}H NMR spectra of quinolines **6a** and **8a** 4
4. ^{1}H and ^{13}C NMR spectra of quinolines **6a-j** and **7a-b** 6
5. References 19

1. Synthesis of 1,3-butadiynamides 1a,b[32]

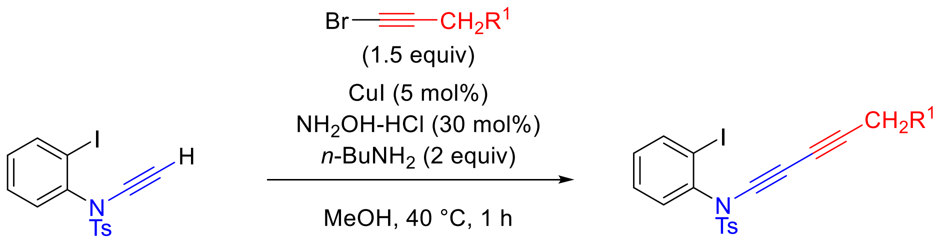

A

1a, R^1 = *n*-Pr
1b, R^1 = $(CH_2)_2OBn$

N-(2-iodophenyl)-4-methyl-*N*-(octa-1,3-diyn-1-yl)benzenesulfonamide (1a)

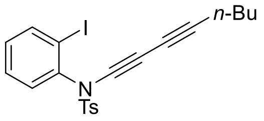

In a two-neck flask equipped with a reflux condenser were introduced under argon atmosphere CuI (30 mg, 0.16 mmol, 5 mol%) and $NH_2OH\cdot HCl$ (66 mg, 0.95 mmol, 30 mol%) in oxygen-

free MeOH (30 mL). To the mixture was added *n*-BuNH$_2$ (0.62 mL, 2 equiv) followed by MeOH (20 mL) and solid *N*-ethynyl-*N*-(2-iodophenyl)-4-methylbenzenesulfonamide (**A**)[33] (1.25 g, 3.2 mmol). The resulting solution was heated at 40 °C and a solution of 1-bromo-1-hexyne (761 mg, 4.73 mmol, 1.5 equiv) in 30 mL of oxygen-free MeOH was added slowly within 25 min. The reaction mixture was further heated at 40 °C for 1 h. Then CH_2Cl_2 and brine were added. The aqueous phase was extracted with CH_2Cl_2 and the combined organic layers were washed with brine, dried ($MgSO_4$), filtered and evaporated under reduced pressure. The product was purified by column chromatography (SiO_2, *n*-pentane/EtOAc 85:15 (v/v)) to afford **1a** as a solid (1.5 g, 3.2 mmol, Yield: 99%). Mp 75–76 °C ($CHCl_3$/*n*-pentane). R_f = 0.60 (*n*-pentane/EtOAc 8:2 (v/v)). ^{1}H NMR (500 MHz, $CDCl_3$) δ 7.88 (dd, ^3J = 7.8 Hz, ^4J = 1.4 Hz, 1 H), 7.76 (d, ^3J = 8.3 Hz, 2 H), 7.36 (d, ^3J = 8.1 Hz, 2 H), 7.31 (m, 1 H), 7.10–7.06 (m, 2 H), 2.48 (s, 3 H), 2.30 (t, ^3J = 7.0 Hz, 2 H), 1.53–1.47 (m, 2 H), 1.44–1.36 (m, 2 H), 0.90 (t, ^3J = 7.3 Hz, 3 H). ^{13}C NMR (125 MHz, $CDCl_3$) δ 145.6 (C), 140.8 (CH), 140.4 (C), 134.4 (C), 131.0 (CH), 130.0 (CH), 129.5 (CH), 129.3 (CH), 128.8 (CH), 99.1 (C), 85.3 (C), 66.6 (C), 64.4 (C), 59.2 (C), 30.4 (CH_2), 22.1 (CH_2), 21.9 (CH_3), 19.5 (CH_2), 13.7 (CH_3). HRMS (ESI+): Calcd for $C_{21}H_{21}INO_2S$ $[M+H]^+$: 478.0338; found: 478.0342.

N-(7-(Benzyloxy)hepta-1,3-diyn-1-yl)-*N*-(2-iodophenyl)-4-methylbenzenesulfonamide (1b)

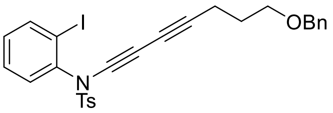

Prepared according to the procedure described for **1a** from **A**[33] (704 mg, 1.8 mmol) and [[(5-bromo-4-pentyn-1-yl)oxy]methyl]benzene (683 mg, 2.7 mmol, 1.5 equiv). The product was purified by column chromatography (SiO_2, *n*-pentane/EtOAc 9:1 (v/v), 8:2 (v/v) to 7:3 (v/v)) to afford **1b** (853 mg, 1.5 mmol, Yield: 83%) as a solid. Mp 76–77 °C. R_f = 0.36 (*n*-pentane/EtOAc 8:2 (v/v)). ^{1}H NMR (600 MHz, $CDCl_3$) δ 7.89 (dd, ^3J = 7.8 Hz, ^4J = 1.3 Hz, 1 H), 7.76 (d, ^3J = 8.3 Hz, 2 H), 7.36 (d, ^3J = 8.2 Hz, 2 H), 7.34–7.31 (m, 5 H), 7.28–7.25 (m, 1 H), 7.10–7.06 (m, 2 H), 4.50 (s, 2 H), 3.55 (t, ^3J = 6.0 Hz, 2 H), 2.48 (s, 3 H), 2.44 (t, ^3J = 7.0 Hz, 2 H), 1.82 (m, 2 H). ^{13}C NMR (150 MHz, $CDCl_3$) δ 145.6 (C), 140.8 (CH), 140.4 (C), 138.5 (C), 134.4 (C), 131.0 (CH), 130.0 (CH), 129.5 (CH), 129.4 (CH), 128.8 (CH), 128.5 (CH), 127.8 (CH), 127.7 (CH), 99.1 (C), 84.5 (C), 73.1 (CH_2), 68.7 (CH_2), 66.7 (C), 64.8 (C), 59.1 (C), 28.6 (CH_2), 21.9 (CH_3), 16.7 (CH_2). HRMS (ESI+): Calcd for $C_{27}H_{24}INO_3S$ $[M]^+$: 569.0522; found: 569.0538.

2. Optimization Table of the reaction conditions of quinoline synthesis

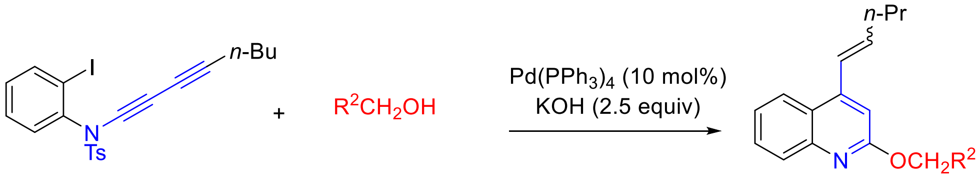

1a **5b,c** **6b,c**

Entry	Alcohol	**5** (equiv)	Time (min)	Solvent	TBAF (equiv)	Product	Yield (%)	E/Z ratio
1	EtOH	**5b** (165)	60	-	-	**6b**	79	97:3
2	EtOH	**5b** (10)	30	THF	-	**6b**	79	97:3
3	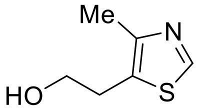	**5c** (10)	60	THF	-	**6c**	-[a]	-
4	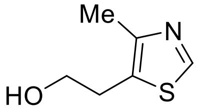	**5c** (20)	60	THF	9.5	**6c**	39	95:5

[a] SM recovered

3. Comparison of the ^{1}H NMR spectra of quinolines 6a and 8a

^{1}H NMR ($CDCl_3$, 500 MHz) spectra of quinolines 6a (top) and 8a (bottom):

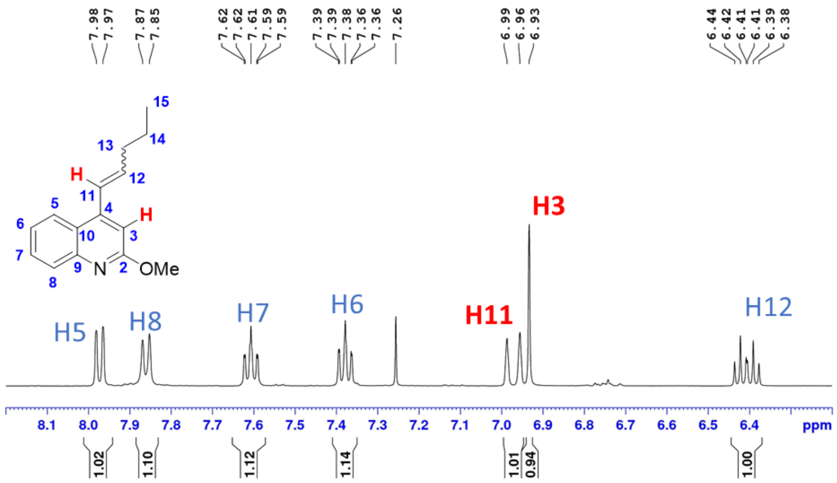

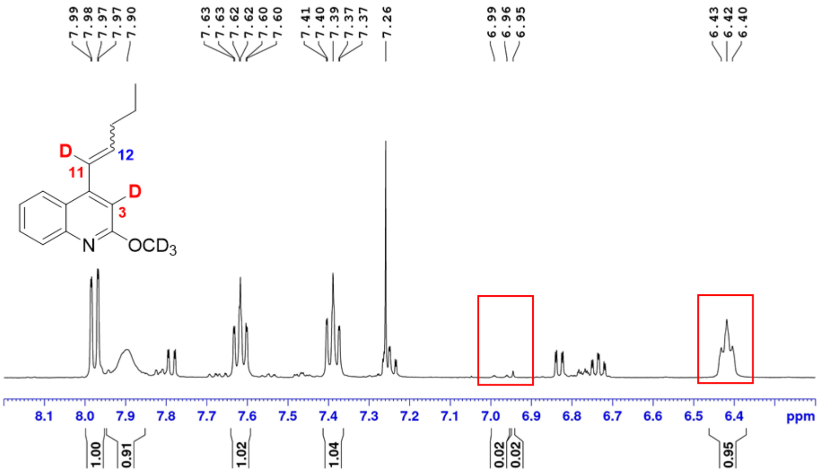

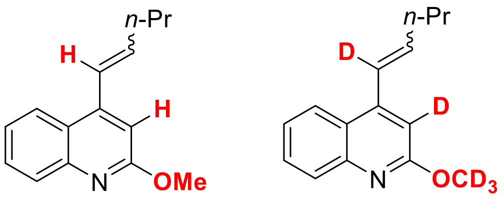

6a **8a**

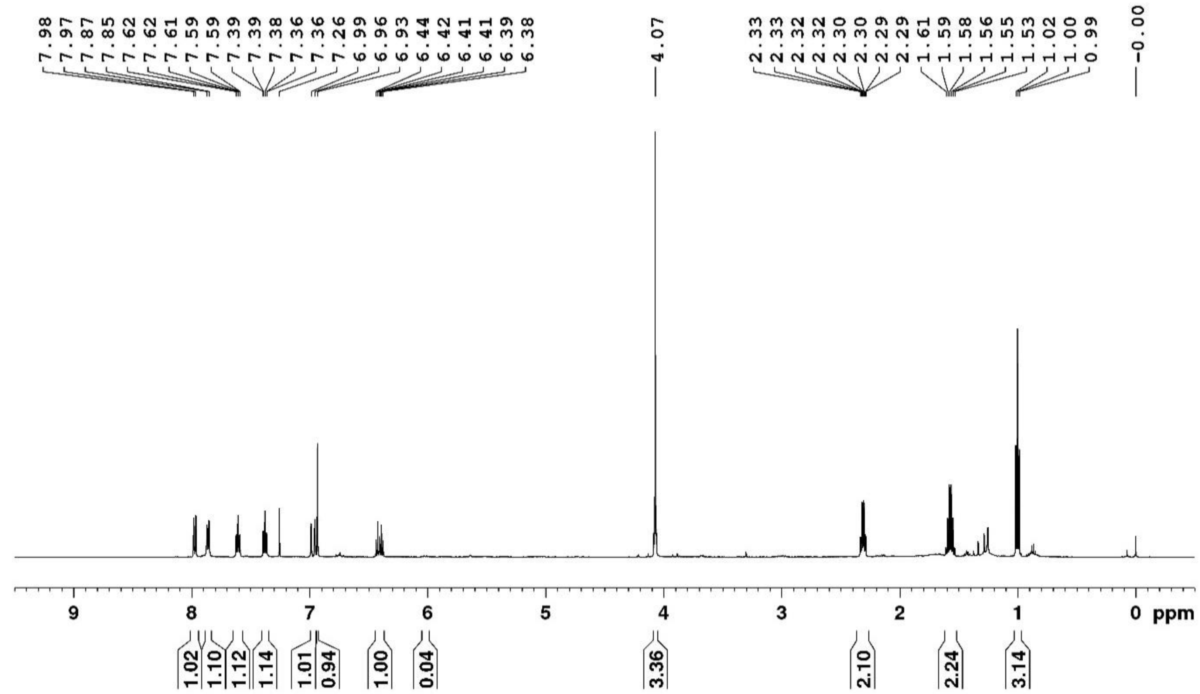

¹H NMR (500 MHz, $CDCl_3$) of **6a**

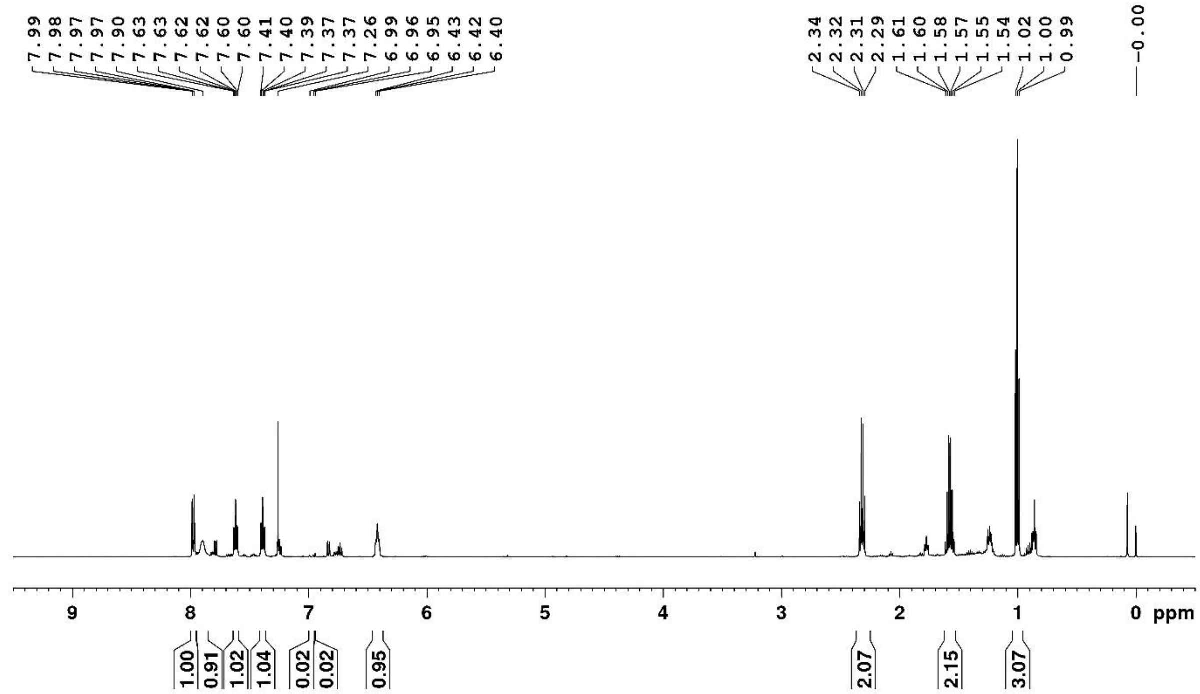

¹H NMR (500 MHz, $CDCl_3$) of **8a**

4. ^{1}H and ^{13}C NMR spectra of quinolines 6a-j and 7a-b

2-Methoxy-4-(pent-1-en-1-yl)quinoline (6a)

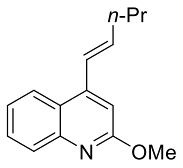

6a (*E*/*Z* 96:4)

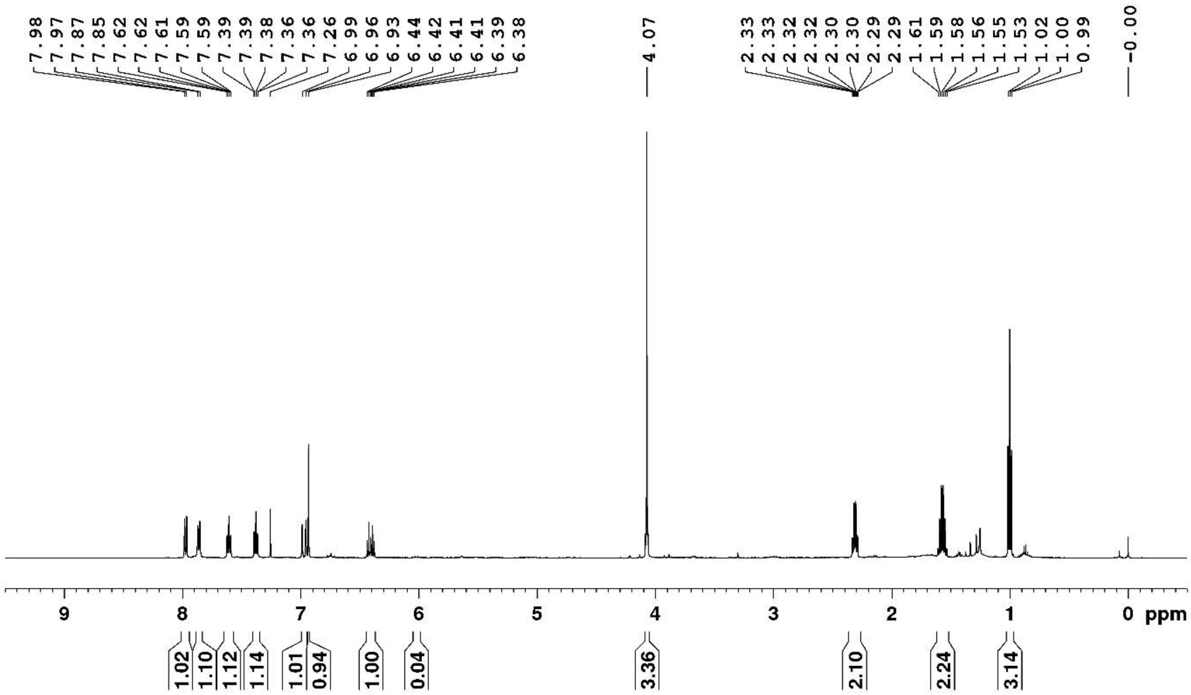

^{1}**H NMR** (500 MHz, $CDCl_3$) of **6a**

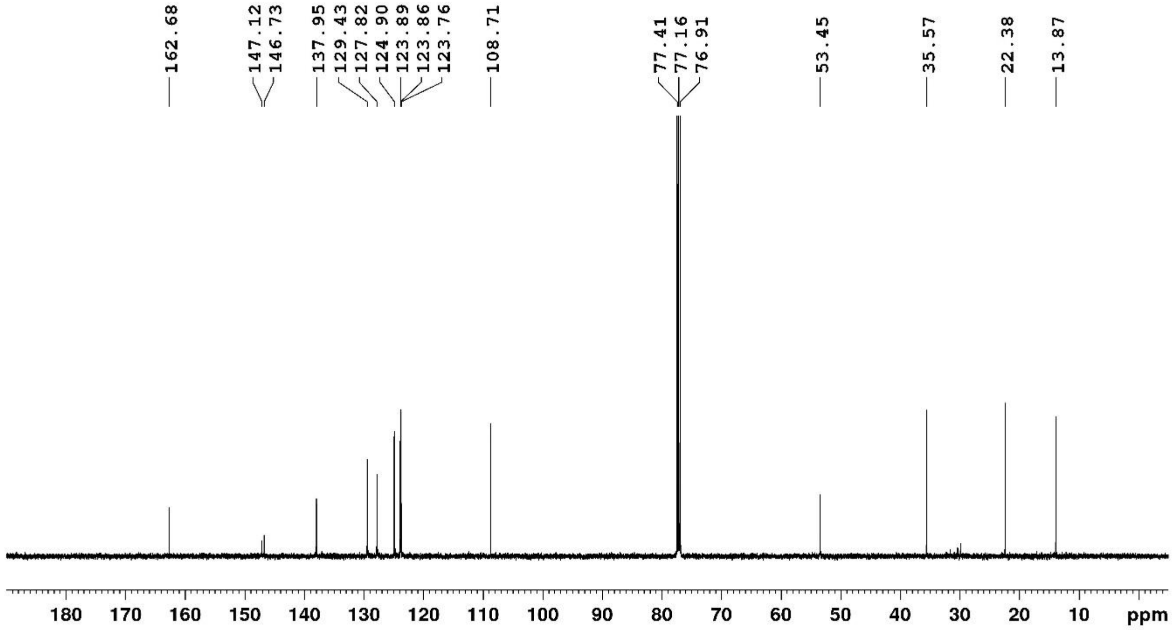

^{13}**C NMR** (125 MHz, $CDCl_3$) of **6a**

2-Ethoxy-4-(pent-1-en-1-yl)quinoline (6b)

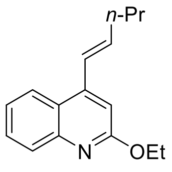

6b (*E*/*Z* 97:3)

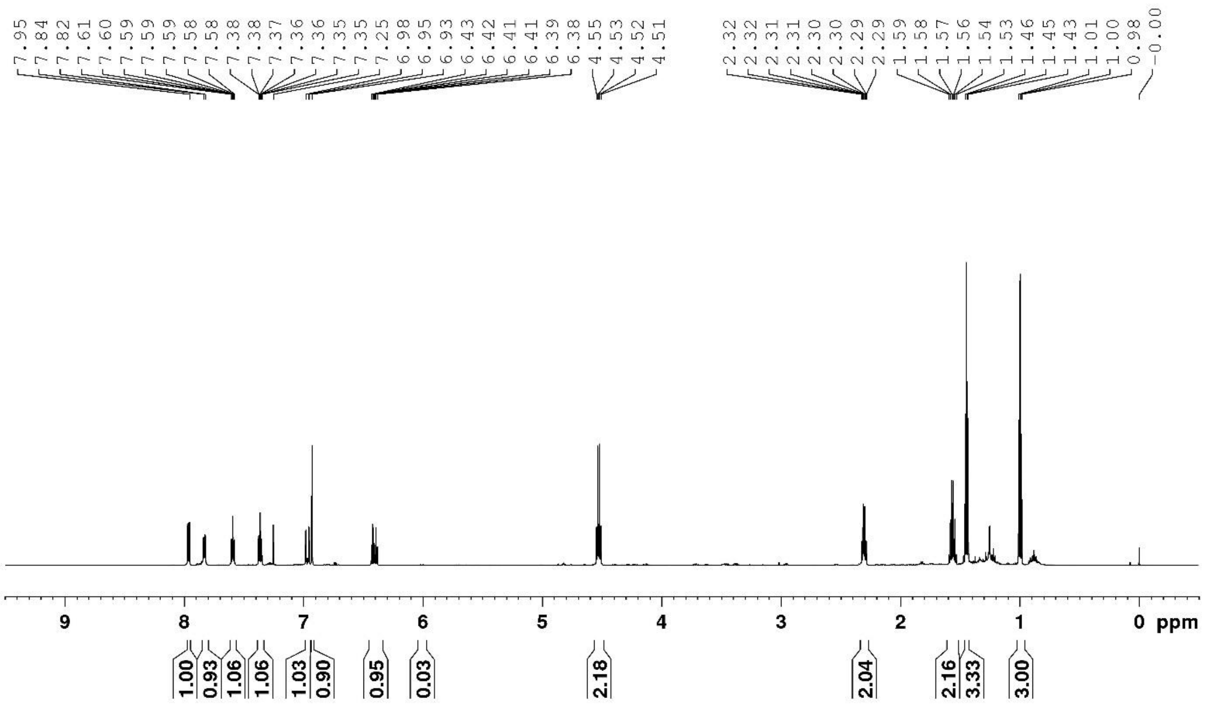

^{1}H NMR (600 MHz, $CDCl_3$) of **6b**

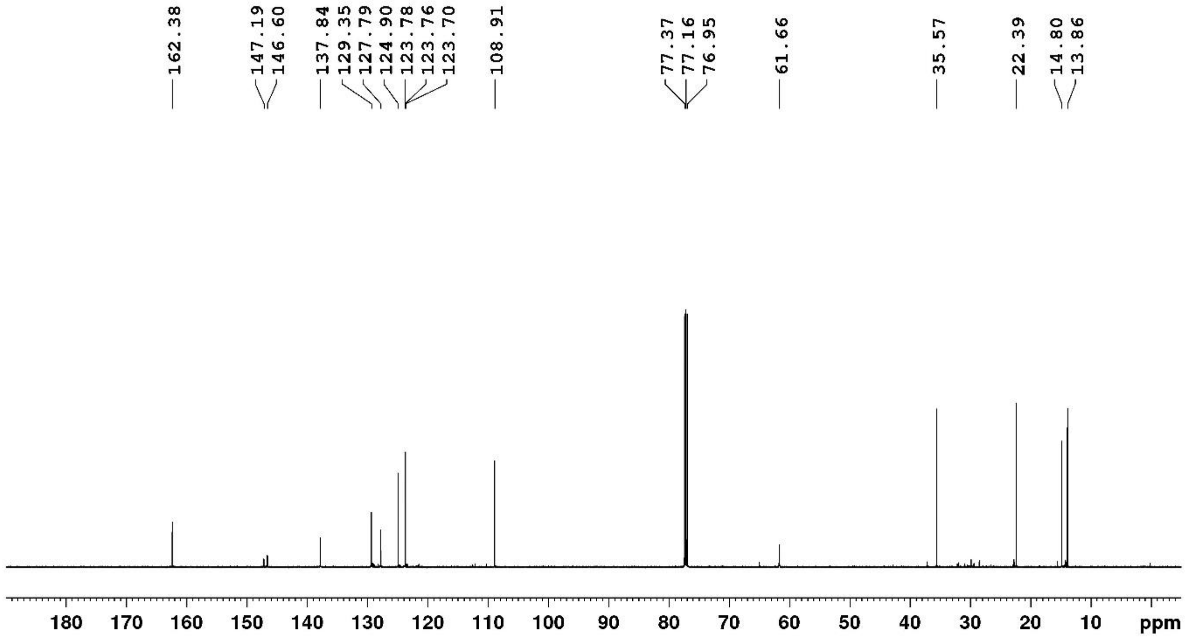

^{13}C NMR (150 MHz, $CDCl_3$) of **6b**

4-Methyl-5-(2-((4-(pent-1-en-1-yl)quinolin-2-yl)oxy)ethyl)thiazole (6c)

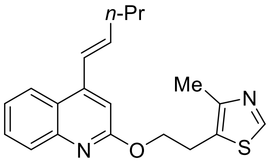

6c (*E*/*Z* 95:5)

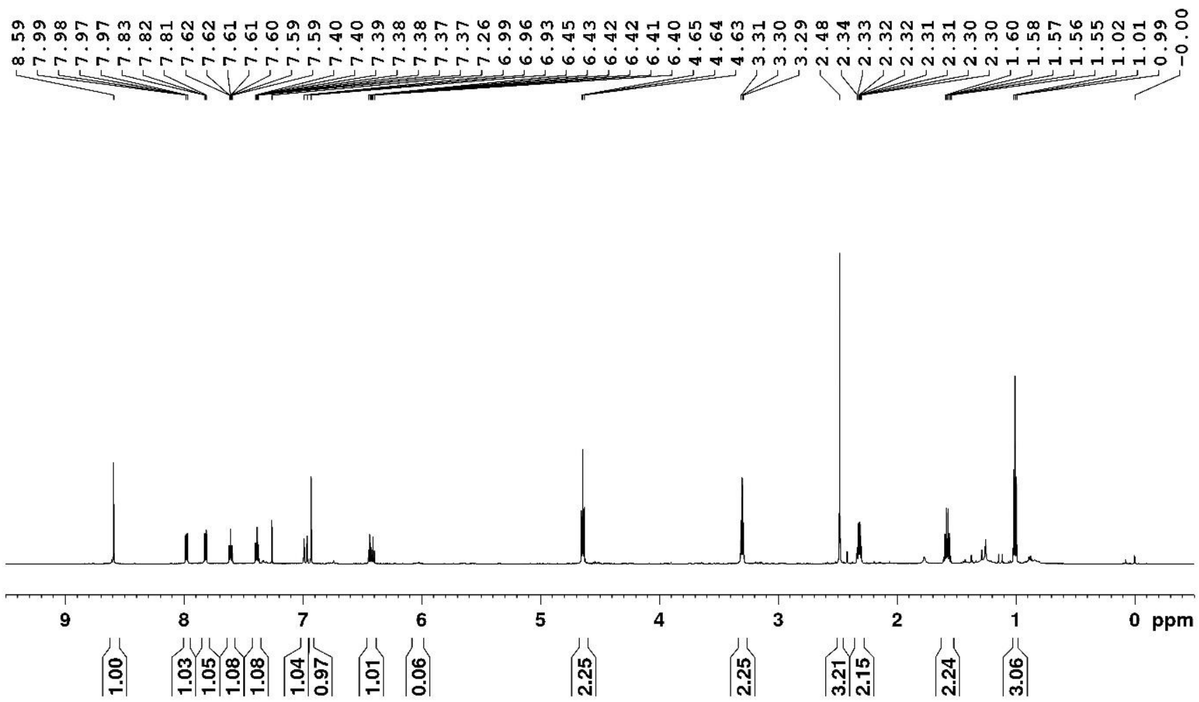

^{1}H NMR (600 MHz, $CDCl_3$) of **6c**

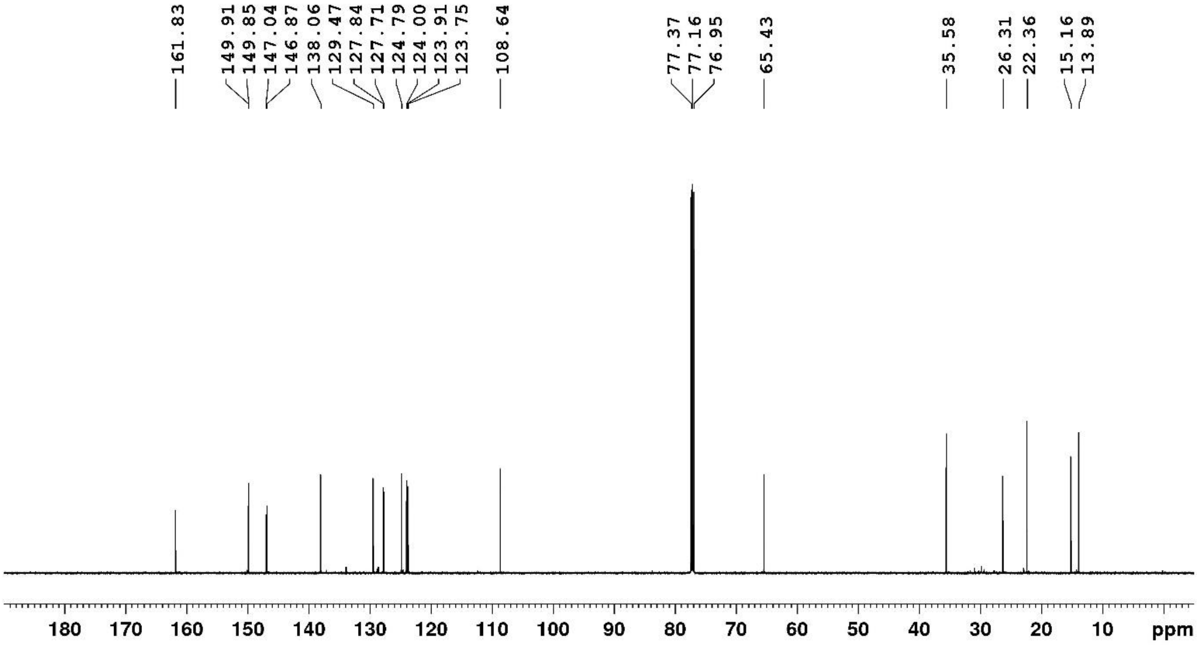

^{13}C NMR (150 MHz, $CDCl_3$) of **6c**

2-((4-(Pent-1-en-1-yl)quinolin-2-yl)oxy)ethanol (6d)

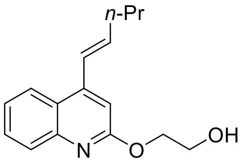

6d (*E*/*Z* 98:2)

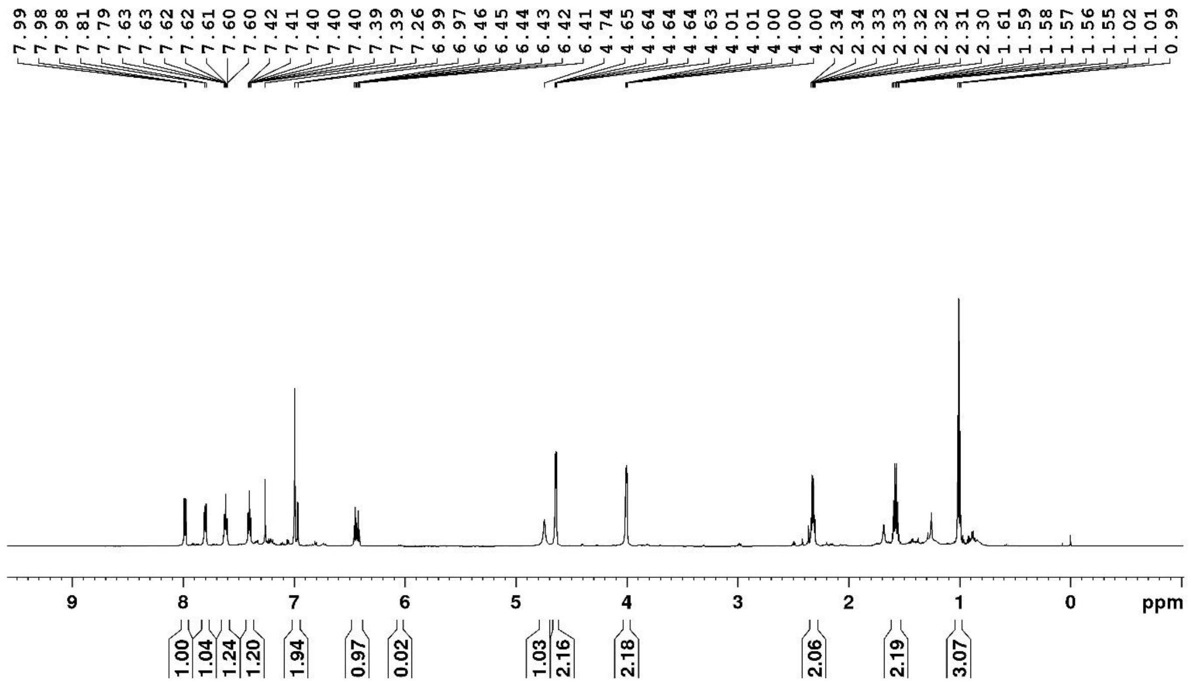

^{1}H NMR (600 MHz, $CDCl_3$) of **6d**

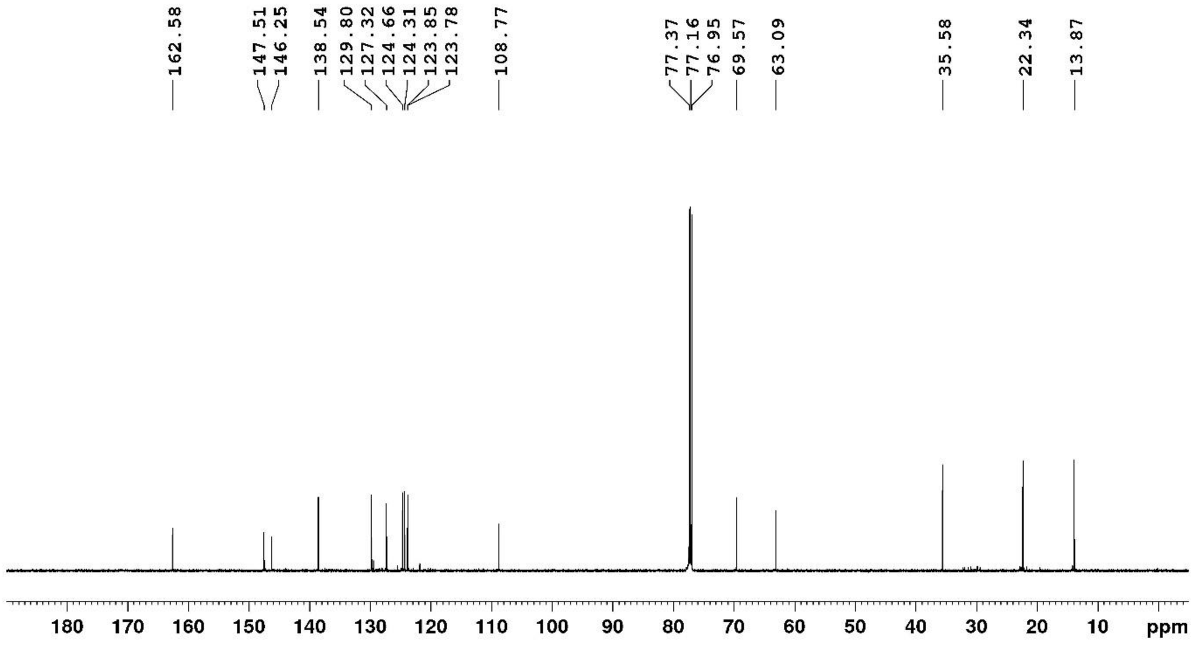

^{13}C NMR (150 MHz, $CDCl_3$) of **6d**

4-((4-(Pent-1-en-1-yl)quinolin-2-yl)oxy)butan-2-ol (6e)

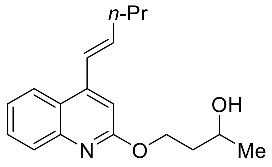

6e (*E*/*Z* 98:2)

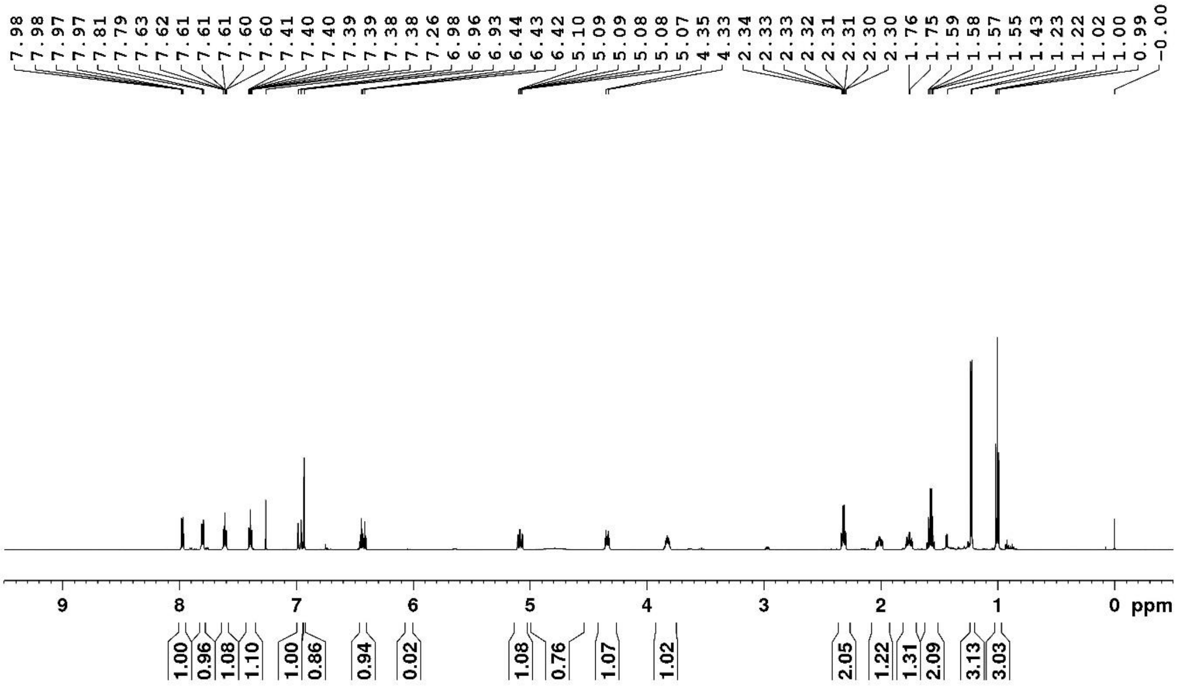

^{1}H NMR (600 MHz, $CDCl_3$) of **6e**

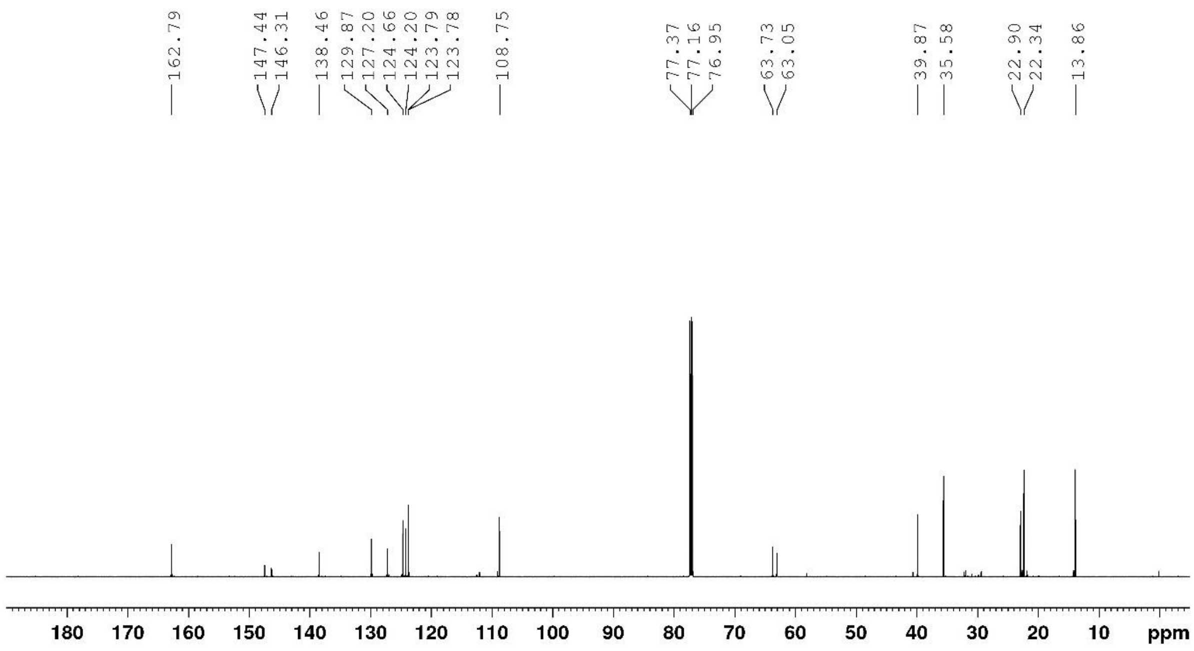

^{13}C NMR (150 MHz, $CDCl_3$) of **6e**

N,N-Dimethyl-2-((4-(pent-1-en-1-yl)quinolin-2-yl)oxy)ethanamine (6f)

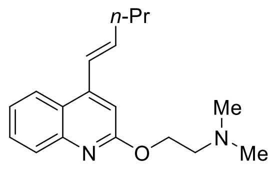

6f (*E*/*Z* 97:3)

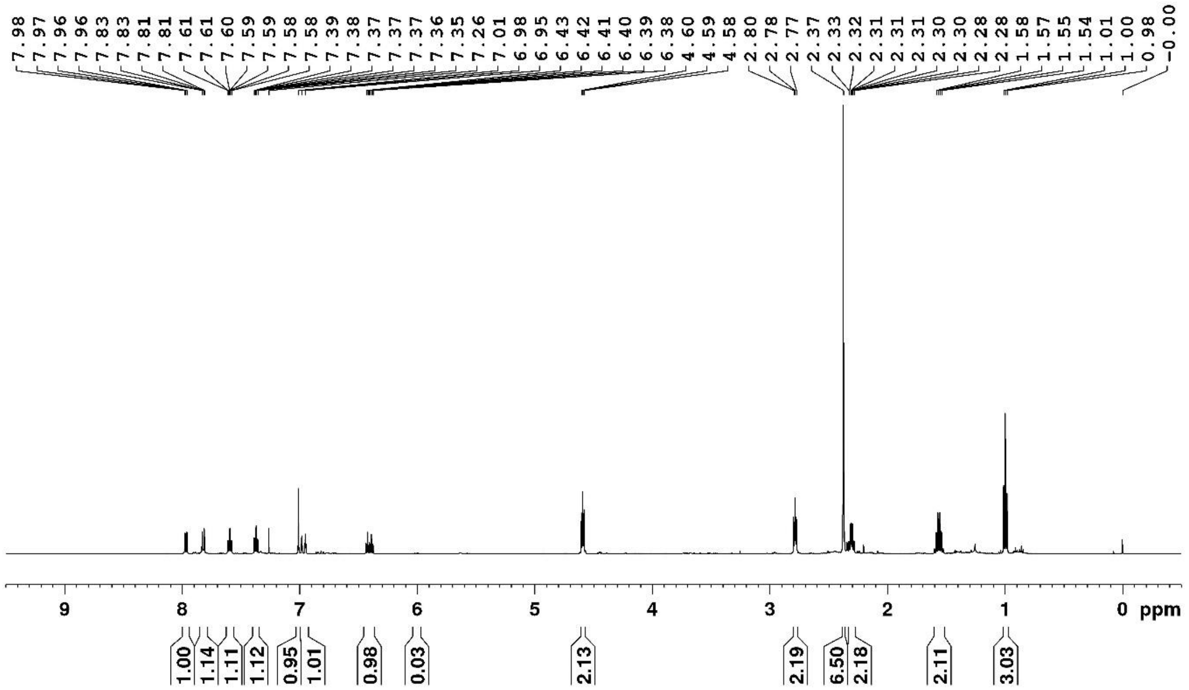

^{1}H NMR (500 MHz, $CDCl_3$) of **6f**

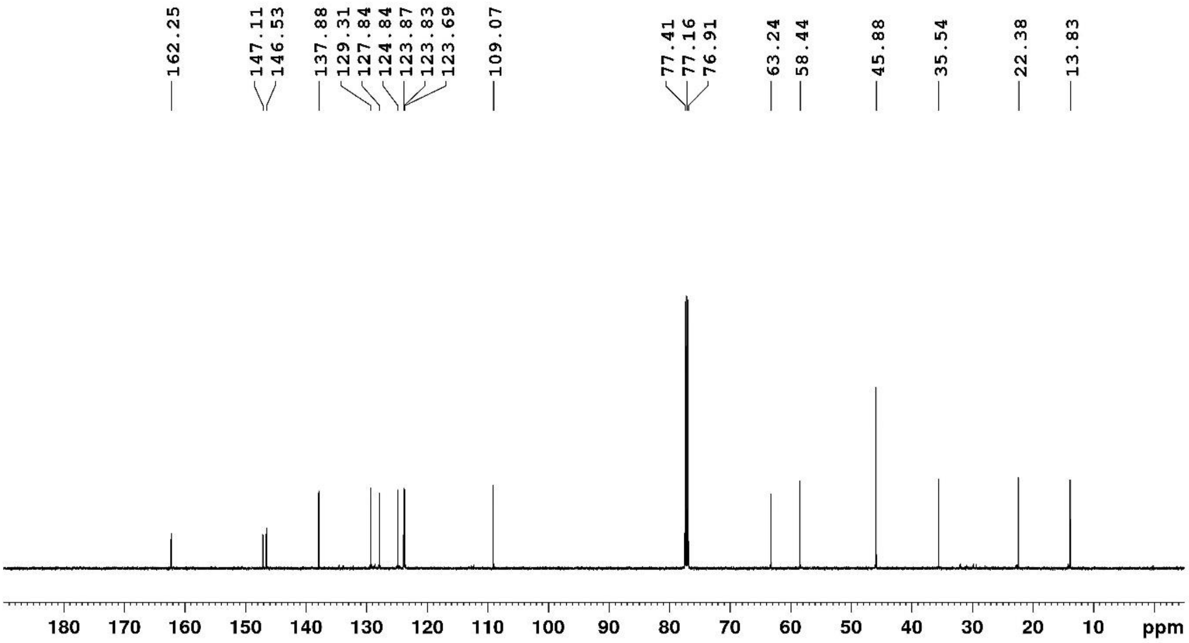

^{13}C NMR (125 MHz, $CDCl_3$) of **6f**

2-((3-Methylbut-3-en-1-yl)oxy)-4-(pent-1-en-1-yl)quinoline (6g)

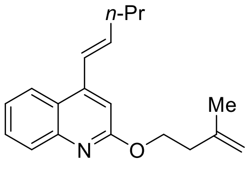

6g (*E*/*Z* 97:3)

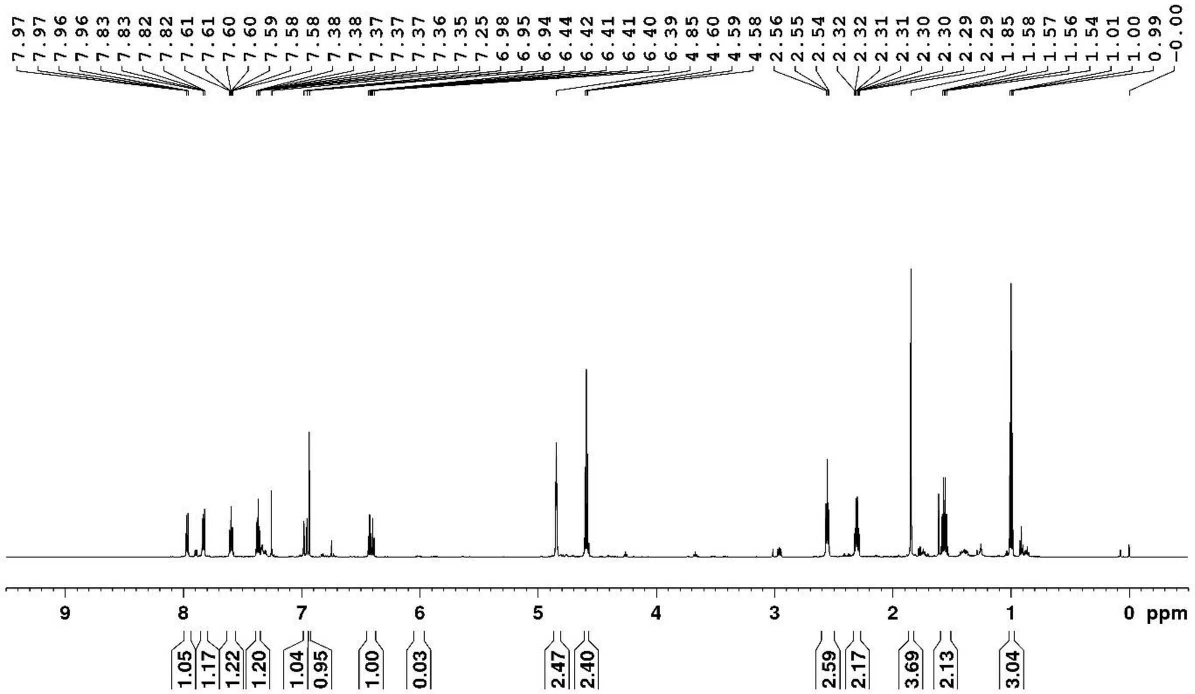

^{1}H NMR (600 MHz, $CDCl_3$) of **6g**

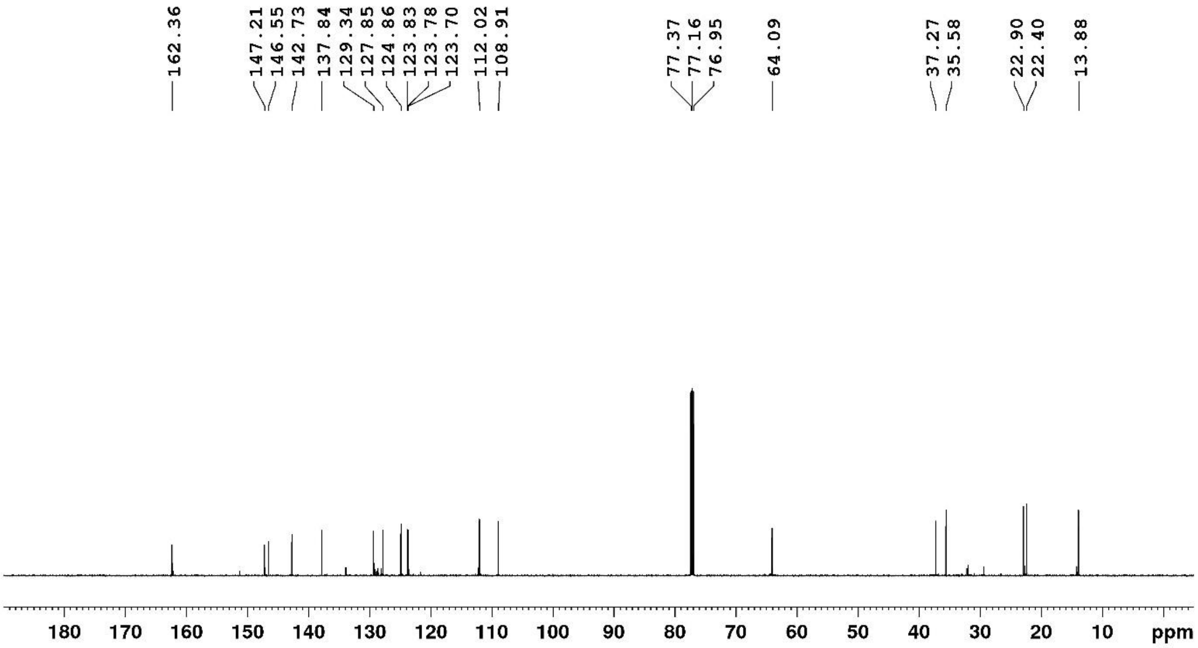

^{13}C NMR (150 MHz, $CDCl_3$) of **6g**

(*E*)-2-(Benzo[d][1,3]dioxol-5-ylmethoxy)-4-(pent-1-en-1-yl)quinoline (6h)

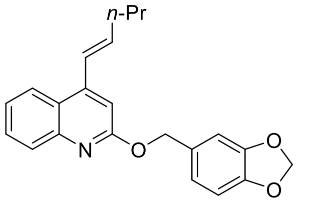

(*E*)-**6h**

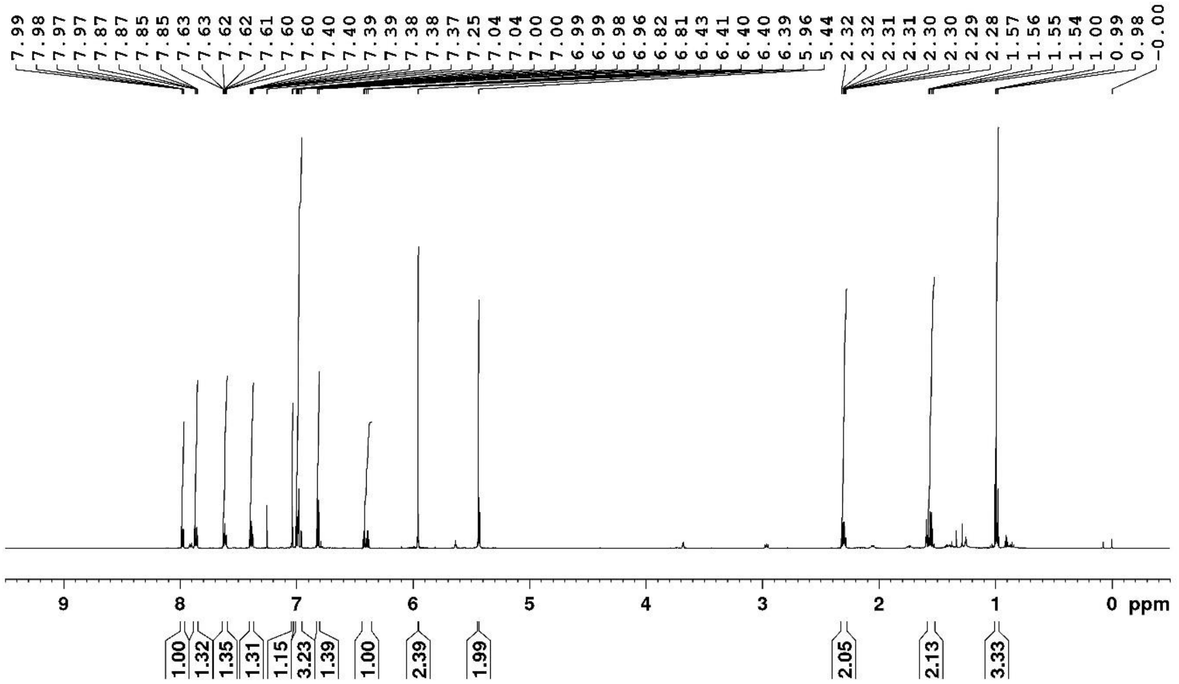

^{1}H NMR (600 MHz, $CDCl_3$) of **6h**

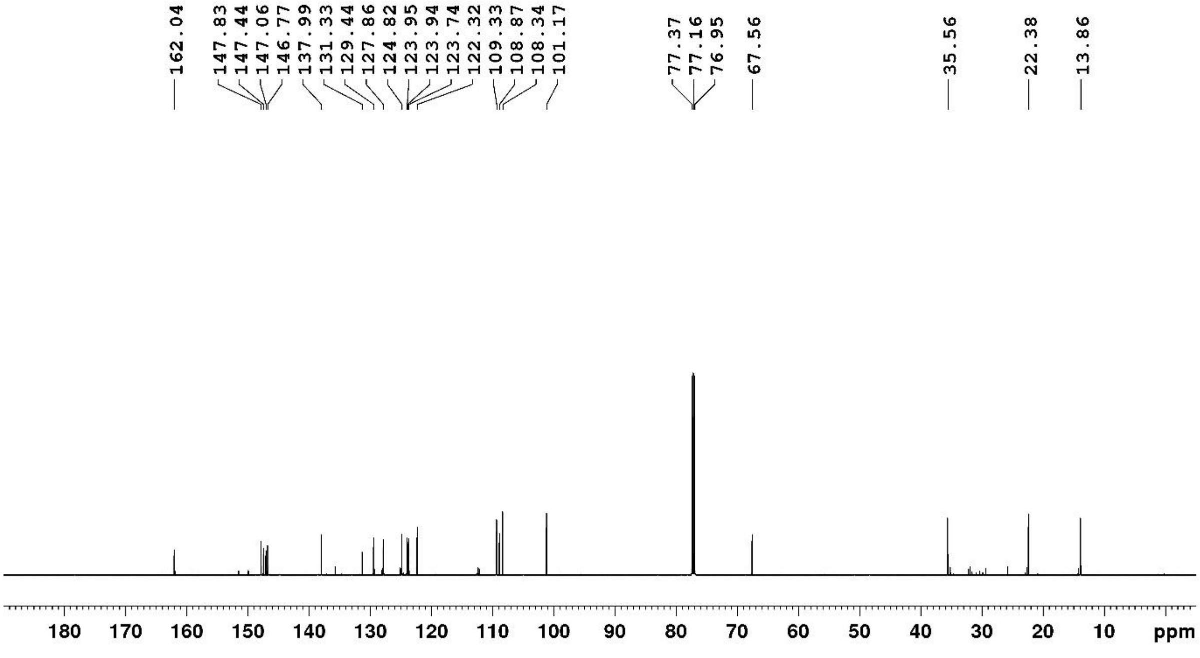

^{13}C NMR (150 MHz, $CDCl_3$) of **6h**

2-(4-Fluorophenyl)ethoxy)-4-(pent-1-en-1-yl)quinoline (6i)

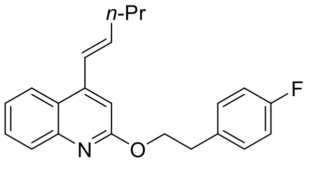

6i (*E*/*Z* 98:2)

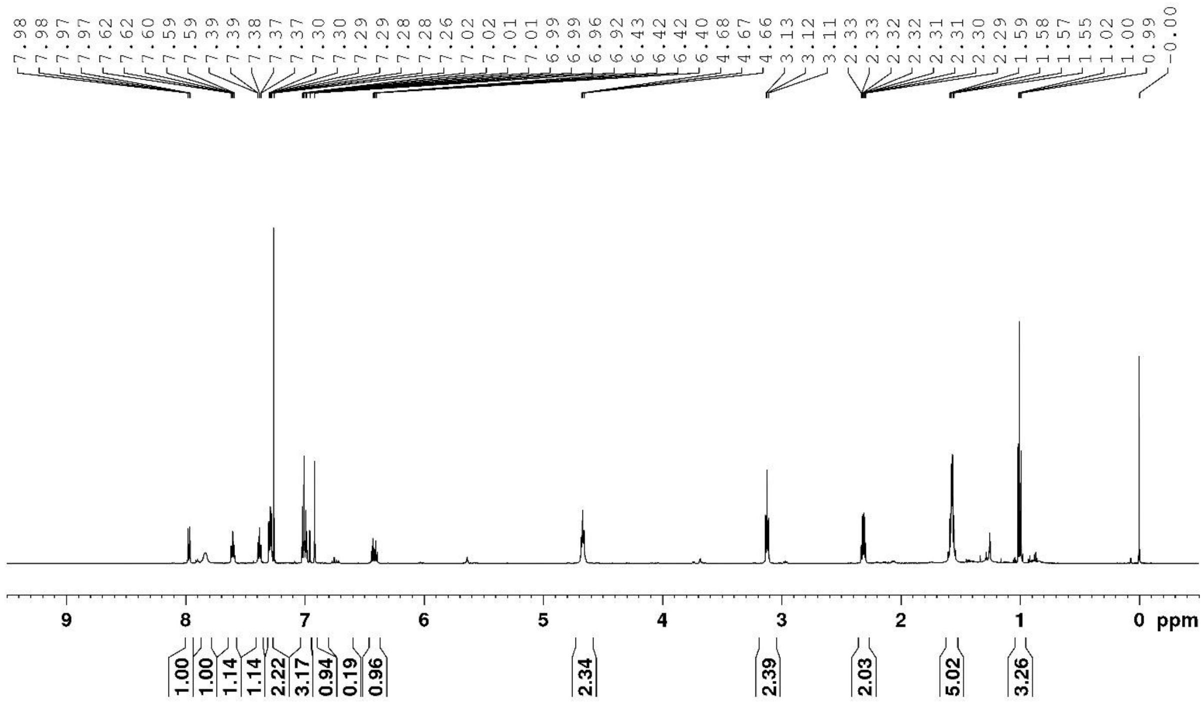

¹H NMR (600 MHz, $CDCl_3$) of **6i**

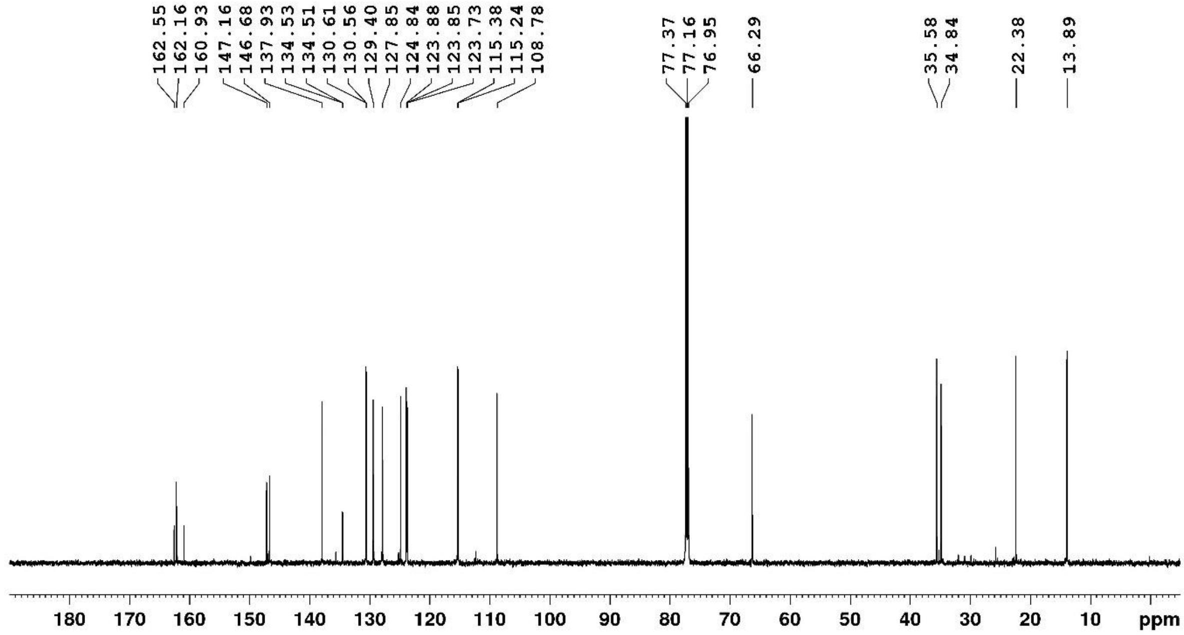

¹³C NMR (150 MHz, $CDCl_3$) of **6i**

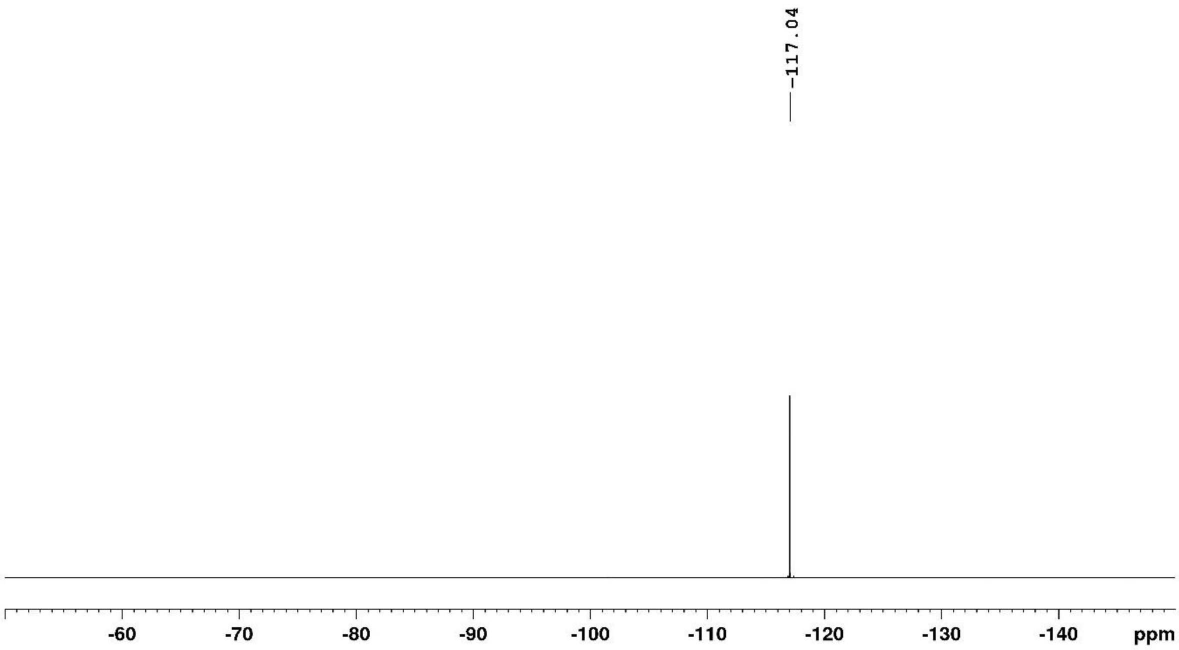

^{19}F{H} NMR (470 MHz, $CDCl_3$) of **6i**

(*E*)-4-(4-(Benzyloxy)but-1-en-1-yl)-2-ethoxyquinoline (6j)

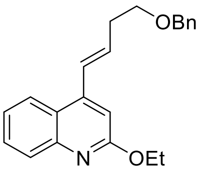

(*E*)-**6j**

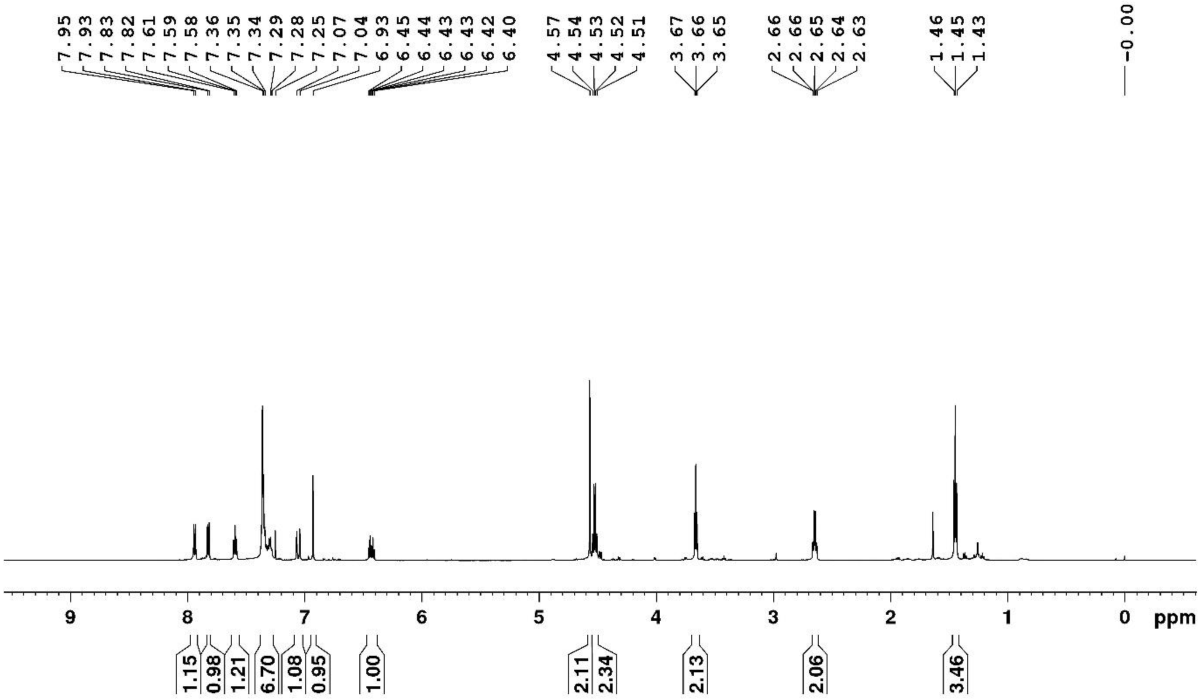

^{1}H NMR (600 MHz, $CDCl_3$) of **6j**

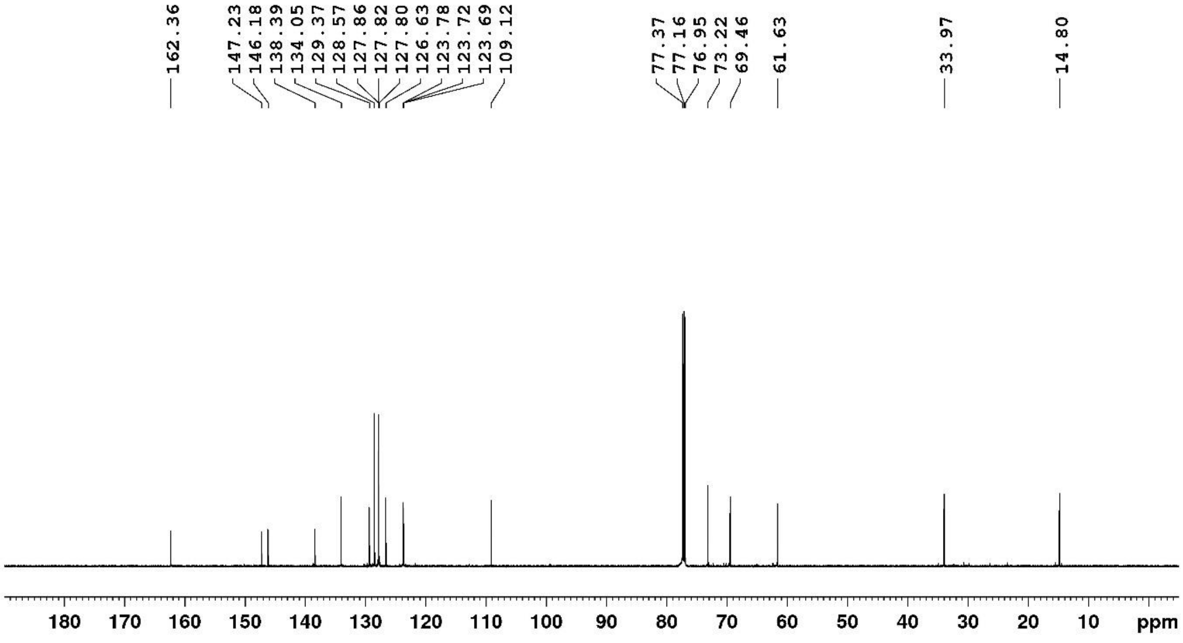

^{13}C NMR (150 MHz, $CDCl_3$) of **6j**

(*E*)-2-Ethoxy-4-(buta-1,3-dien-1-yl)quinoline (7a)

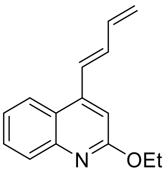

(*E*)-**7a**

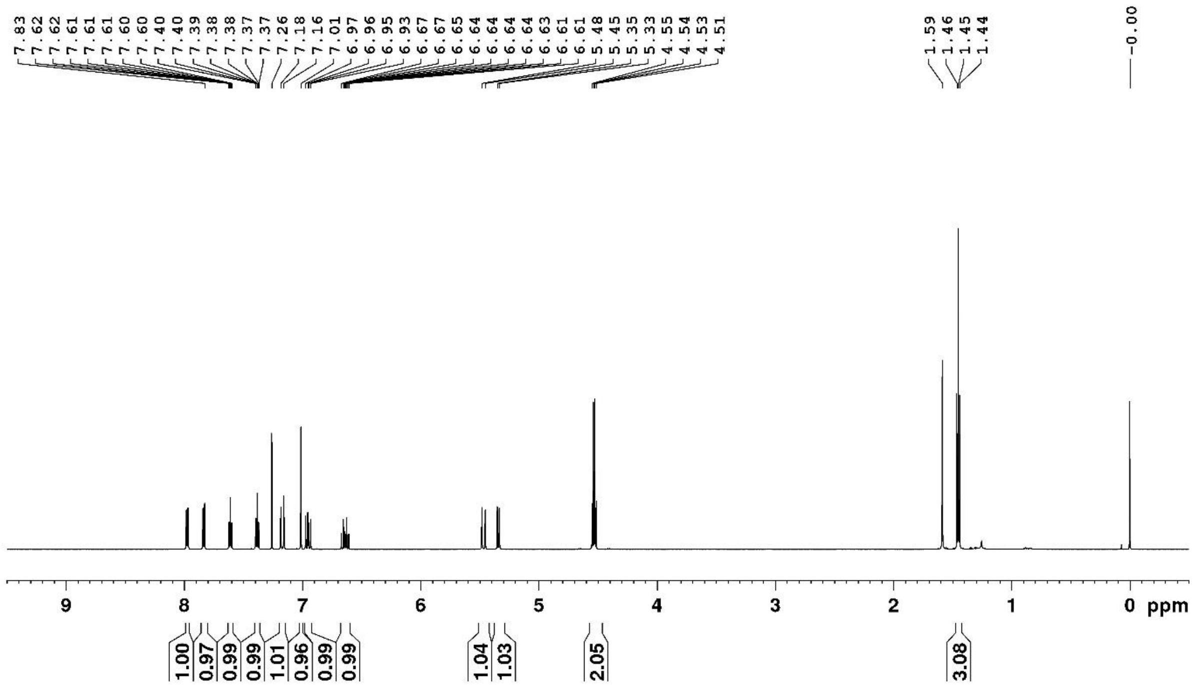

^{1}H NMR (600 MHz, $CDCl_3$) of **7a**

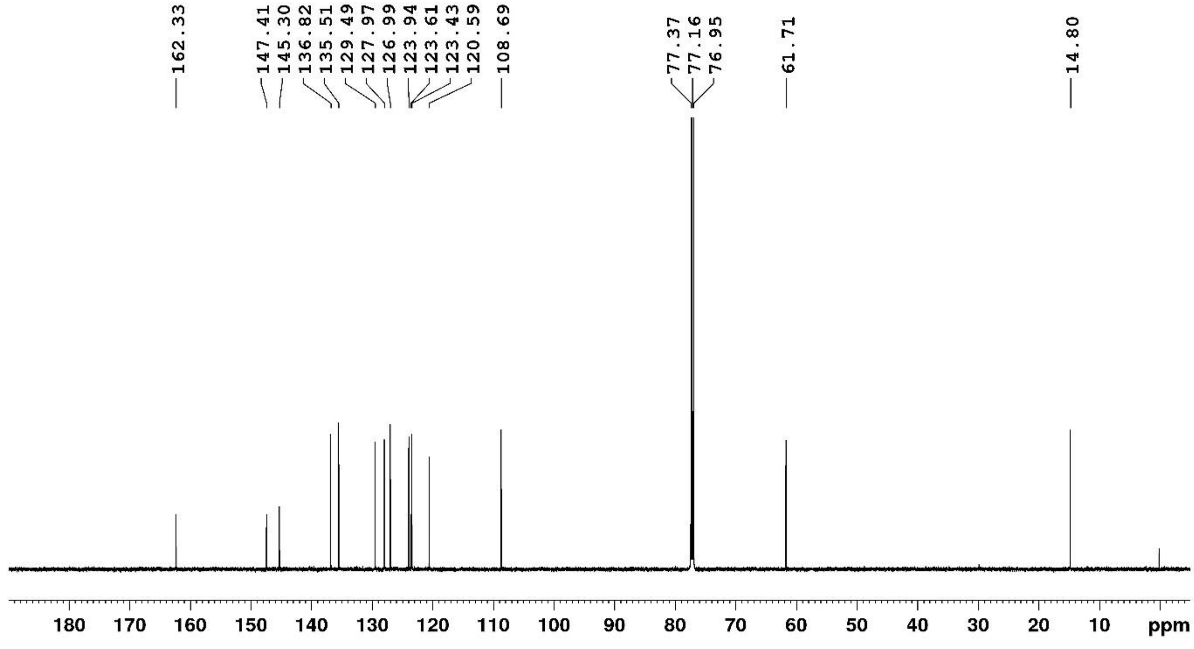

^{13}C NMR (150 MHz, $CDCl_3$) of **7a**

(*E*)-5-(2-((4-(buta-1,3-dien-1-yl)quinolin-2-yl)oxy)ethyl)-4-methylthiazole (7b)

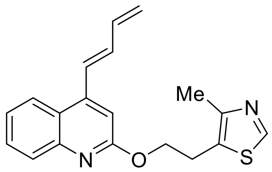

(*E*)-**7b**

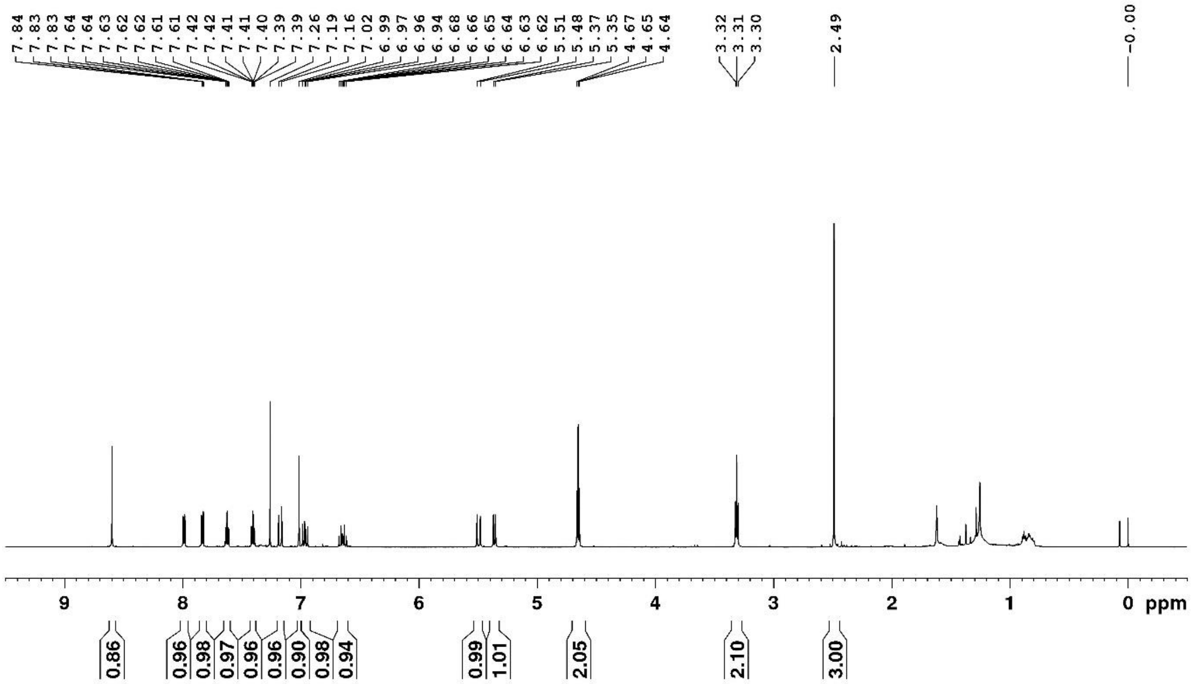

^{1}H NMR (600 MHz, $CDCl_3$) of **7b**

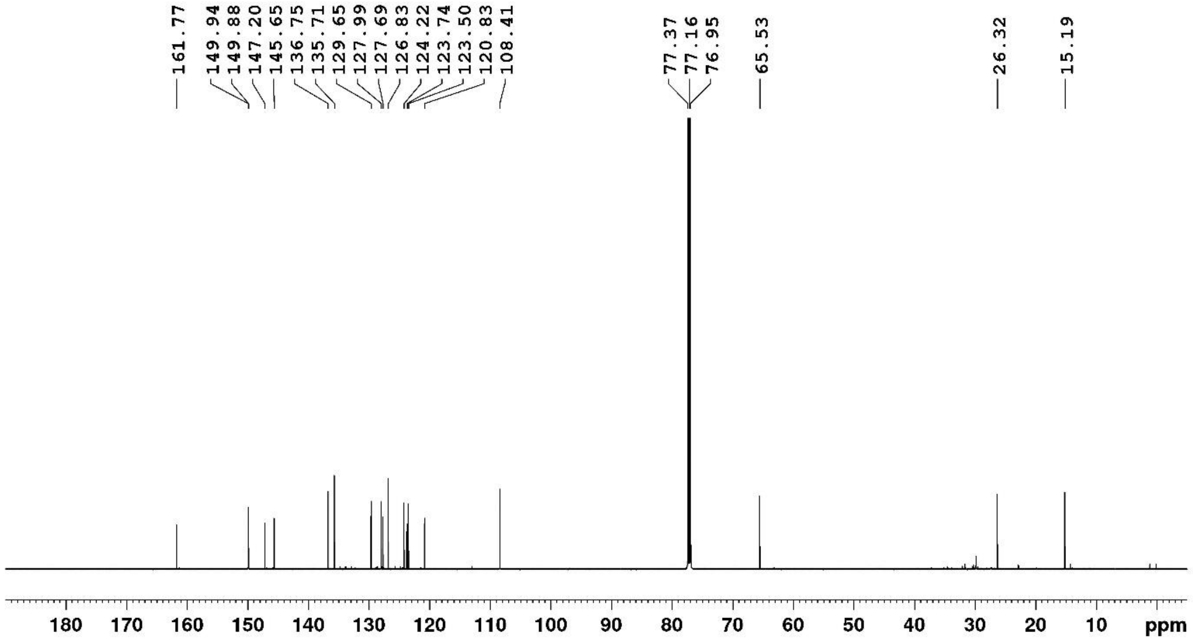

^{13}C NMR (150 MHz, $CDCl_3$) of **7b**

5. References

[32] Lenko, I.; Mamontov, A.; Alayrac, C.; Legay, R.; Witulski, B. Media-Driven Pd-Catalyzed Reaction Cascades with 1,3-Diynamides Leading Selectively to Either Indoles or Quinolines. *Angew. Chem. Int. Ed.* **2021**, *60*, 22729–22734.

[33] Witulski, B.; Alayrac, C.; Tevzadze-Saeftel, L. Palladium-Catalyzed Synthesis of 2-Aminoindoles by a Heteroanulation Reaction. *Angew. Chem. Int. Ed.* **2003**, *42*, 4257–4260.
